# Characteristics and whole-genome analysis of a novel *Pseudomonas syringae* pv. *tomato* bacteriophage D6 isolated from a karst cave

**DOI:** 10.1007/s11262-024-02064-9

**Published:** 2024-04-09

**Authors:** Qingshan Wu, Ni An, Zheng Fang, Shixia Li, Lan Xiang, Qiuping Liu, Leitao Tan, Qingbei Weng

**Affiliations:** 1 School of Life Sciences, Guizhou Normal University, Guiyang, 550025 People’s Republic of China; 2 Qiannan Normal College for Nationalities, Duyun, 558000 People’s Republic of China

**Keywords:** *Pseudomonas syringae*, Plant pathogen, *Caudoviricetes*, Bacteriophage, Whole genome analysis, Biocontrol, Karst cave

## Abstract

*Pseudomonas syringae* is a gram-negative plant pathogen that infects plants such as tomato and poses a threat to global crop production. In this study, a novel lytic phage infecting *P. syringae* pv. *tomato* DC3000, named phage D6, was isolated and characterized from sediments in a karst cave. The latent period of phage D6 was found to be 60 min, with a burst size of 16 plaque-forming units per cell. Phage D6 was stable at temperatures between 4 and 40 °C but lost infectivity when heated to 70 °C. Its infectivity was unaffected at pH 6–10 but became inactivated at pH ≤ 5 or ≥ 12. The genome of phage D6 is a linear double-stranded DNA of 307,402 bp with a G + C content of 48.43%. There is a codon preference between phage D6 and its host, and the translation of phage D6 gene may not be entirely dependent on the tRNA library provided by the host. A total of 410 open reading frames (ORFs) and 14 tRNAs were predicted in its genome, with 92 ORFs encoding proteins with predicted functions. Phage D6 showed low genomic similarity to known phage genomes in the GenBank and Viral sequence databases. Genomic and phylogenetic analyses revealed that phage D6 is a novel phage. The tomato plants were first injected with phage D6, and subsequently with *Pst* DC3000, using the foliar spraying and root drenching inoculum approach. Results obtained after 14 days indicated that phage D6 inoculation decreased *P. syringae-*induced symptoms in tomato leaves and inhibited the pathogen’s growth in the leaves. The amount of *Pst* DC3000 was reduced by 150- and 263-fold, respectively. In conclusion, the lytic phage D6 identified in this study belongs to a novel phage within the *Caudoviricetes* class and has potential for use in biological control of plant diseases.

**Supplementary Information:**

The online version contains supplementary material available at 10.1007/s11262-024-02064-9.

## Introduction

Pathogen infection is one of the major reasons for crop yield reduction [1]. Several strategies have been employed to combat plant bacterioses, including the use of bactericides with varying properties (from metal-containing organic compounds to plant extracts), seed sterilization and coating with growth-promoting bacteria, and the modification fields to limit soil-borne pathogens patho [2]. However, these strategies are not always effective and can induce pathogen resistance and further environmental contamination if used extensively their widespread use when [3]. *Pseudomonas syringae* is a bacterial plant pathogen that infects over 180 plant species and causes significant economic losses in global agriculture [4]. Many globally important crops are susceptible to *P. syringae*, making it one of the most economically destructive pathogens [5, 6]. *P. syringae* pv. *tomato* (*Pst*), a specific strain of *P. syringae,* infects tomato and *Nicotiana benthamiana,* causing bacterial speck disease and leading to severe yield reductions in both field and greenhouse settings [2].

Bacteriophages (phages) are viruses that specifically infect or lyse archaea or eubacteria [7]. Phages offer several advantages over antibiotics and other antibacterial agents. For instance, they are highly specific in lysing host bacteria, can self-dose replication by lysing host bacteria, and can kill the host without disrupting the normal microbial flora, thereby preventing bacterial disturbance and secondary infections [8]. Therefore, the use of lytic phages to kill host bacteria in phage biocontrol has gained increasing attention as a potential treatment option for bacterial infections [6, 9].

*Pseudomonas* phages have been isolated from a variety of environmental sources, including sewage [10], soil [6], lakes [11], rivers [12], and wastewater [13], and have been successfully applied in biological control. For example, the biocontrol agent phage Eir4 has been found to be efficient against *Pst* in vitro [2]. Additionally, phage PPPL-1 was reported to effective in controlling kiwifruit canker caused by *P. syringae* pv. *actinidiae* [14]. Bacteriophages have also been successfully used in the treatment of chronic otitis [15], respiratory tract infections [16], and trophic ulcers caused by *P. aeruginosa* and other bacteria [17]. The development of lytic bacteriophages for biocontrol and other applications is essential.

In this work, a novel phage named D6 was isolated from sediments in a karst cave. This phage was able to lyse and infect *Pst* DC3000. The morphology, biological properties, genomic characteristics, and biocontrol efficiency in planta of phage D6 were described. The results of this research will contribute to a better understanding of plant pathogenic phage and aid in the development of safe and effective phage preparations for the biocontrol of *P. syringae.* Additionally, this work provides valuable information for further research into the mechanism of host-phage interactions.

## Materials and methods

### Bacteria culture

*Pst* DC3000 strain was used as the bacterial host and was stored in 25% (v/v) glycerol at − 80 °C in the laboratory. The strain was cultured in King’s B (KB) medium at 28 °C, and cells in exponential phase were prepared for phage isolation, enrichment, and plaque assay. Sediment samples for phage isolation were collected in November 2021 from a karst cave in Libo County, Guizhou Province, China, as described previously [18].

### Phage isolation and purification

Thirty gram of the sediment was mixed with 270 mL of sterile distilled water. The supernatant was then filtered through a 0.22 μm pore size filter, and 100 μL of the filtrate was added to 5 mL *Pst* DC3000 culture. The mixture was incubated overnight at 28 °C and 160 rpm. The lysate was centrifuged at 12,000 × *g* for 1 min, and the supernatant was filtered through a 0.22 μm pore size filter. The filtrate was diluted with sodium chloride–magnesium sulfate (SM) buffer and mixed with 1 mL of *Pst* DC3000 culture. After incubating at room temperature for 5 min, the mixture was poured onto double-layer agar for plaque assay [19]. Individual plaques were selected and subjected to at least three consecutive plaque assays to obtain clear and unique phage plaques.

The phage lysates were precipitated with 15% (w/v) PEG 8000 and 0.5 M NaCl overnight at 4 °C, centrifuged at 12,000 × *g* for 20 min, the phage particle precipitate was resuspended in SM buffer [20]. Phage titers were determined by double-layer agar method [21].

### Electron microscopy

Purified phages were loaded onto a carbon coated copper grid, negatively stained with 2% (w/v) potassium phosphotungstate, and visualized and characterized using transmission electron microscopy (Hitachi HT7700, Tokyo, Japan) at an accelerating voltage of 100 kV [22].

### Host range and efficiency of plating (EOP)

The phage host range was determined using 11 strains of *Pseudomonas* isolated from the karst cave in our laboratory. Briefly, 10 μL of the phage suspension (10^8^ PFU/mL) was spotted onto KB plate freshly inoculated with *Pseudomonas* lawn, and incubated for 12 h at 28 °C. All the *Pseudomonas* isolates sensitive to phage D6 in the spot test were selected for further determination of EOP using the double-layer agar method [10].

### SDS-PAGE analysis of phage proteins

Briefly, phage D6 (10^8^ PFU/mL) was mixed with 100 mL of *Pst* DC3000 culture (OD_600nm_ = 0.2) at an MOI of 0.001 and incubated for 6 h at 28 °C and 180 rpm/min. DNase I and RNase A were added to the cell lysate to a final concentration of 1 μg/mL and incubated at 37 °C for 1 h. After 5.84 g NaCl was added to the cell lysate to dissolve, the mixture was incubated on ice for 1 h and then centrifuged at 11,000 × *g* for 10 min at 4 °C. PEG 8000 (10% w/v) was added to the supernatant and dissolved by slow stirring. After the mixture was cooled on ice for 5 h, the mixture was then centrifuged at 11,000 × *g* for 10 min at 4 °C. The phage pellet was suspended in 1.6 mL of SM buffer, then 1.6 mL of chloroform was added and vortex for 30 s. The mixture was centrifuged at 5,000 × *g* for 10 min at 4 °C. The upper phase was extracted again with an equal volume of chloroform, and the upper phase after centrifugation was collected, and analyzed for phage structural proteins by SDS-PAGE. A sample of 15 μL was mixed with an equal volume of loading buffer (10% SDS, 0.1% β-mercaptoethanol, 0.5% bromophenol blue, 0.5 M *Tris*-HCl, pH 6.8) and then heated over a metal bath at 95 °C for 5 min. The sample was then separated by SDS-PAGE (12% acrylamide gel) and visualized after Coomassie brilliant blue R250 (Sigma) staining.

### Multiplicity of infection (MOI) and one step growth curve

MOI refers to the ratio of plaque-forming units (PFUs) to colony-forming units (CFUs) [3]. After determining the phage titer and cell count of *Pst* DC3000 suspension, the phage was mixed with the *Pst* DC3000 suspension in proportion (10, 1, 0.1, 0.01, 0.001, and 0.0001). The phage titers were determined and the ratio producing the highest titer was considered to be the optimum MOI for the phage [21]. The experiments were performed in triplicate for each proportion.

A one step growth curve was undertaken as previously described [22]. *Pst* DC3000 was infected with phage at an MOI of 0.001, and 400 µL of infected cultures were collected every 30 min for 7 h post-infection, respectively. Phage titers were determined.

### Thermal, pH, chloroform and ultraviolet (UV) stability

The thermal stability of the phage was determined by incubating 10^8^ PFU/mL of phage in a water bath at temperatures of 4, 20, 30, 40, 50, 60, and 70 °C for 1 h, respectively. The titers were then measured. To determine the pH stability of the phage, 100 µL of phage suspension (10^8^ PFU/mL) was mixed with 900 µL SM buffer (the pH value was adjusted to between 3 and 12 using 1 mol/L HCl or 1 mol/L NaOH). The phage titers were measured after incubation for 1 h at 28 °C. To determine the sensitivity of the phage to chloroform, after mixing the phage (10^8^ PFU/mL) with or without 25% chloroform, the mixture was mixed upside down and incubated at room temperature for 10 min. The titer was then measured. The effect of UV irradiation on the phage was determined by exposing the phage suspension to ultraviolet (UV) light (50 cm, 30 W) for different periods of time (0, 20, 40, 60, 80, 100, and 120 min), and the phage titer was measured. All experiments were carried out in triplicate.

### Adsorption assay

To determine the adsorption of the phage, phage D6 and *Pst* DC3000 were mixed at an MOI of 0.1 and incubated at 28 °C and 160 rpm. Samples of 300 μL were collected every 5 min for up to 30 min and immediately centrifuged at 10,000 × *g* for 1 min at 4 °C. The titer of the free phages was measured. Adsorption = (1 − titers of free phages / titers of initial phages) × 100%.

### Bacteriostasis test in vitro

One hundred μL of phage D6 and 100 μL of *Pst* DC3000 (OD_600_ = 0.3) were mixed at various MOI (100, 10, 1, 0.1, 0.01, 0.001) in a 96-well plate and incubated at 28 °C and 180 rpm. KB medium was used as a control instead of the phage. Absorbance was measured at OD_600_ every 2 h for up to 26 h.

### Genomic sequencing and annotation

Phage genomic DNA was extracted using the viral genome extraction kit (Takara, Japan) following the manufacturer's instructions. The concentration and quality of DNA were determined at 260 nm and 280 nm using Nanodrop spectrophotometry. DNA libraries were then constructed using the TruSeqTM DNA Sample Prep Kit (Illumina, San Diego, CA, USA) and sequenced in paired-end model using the Illumina NovaSeq platform by Personalbio (Shanghai Personal Biotechnology Co., Ltd, China).

The obtained FastQ raw reads were trimmed of adaptors and low-quality bases and short reads were filtered using AdapterRemoval v2.1.3 [23]. The corrected sequences were assembled into contigs by de novo assembly using A5-MiSeq v20160825 and SPAdes v3.12.0 [24, 25]. Subsequently, the contigs were further assembled into complete genome sequences for the phage using Blast v2.12.0, MUMmer v3.1 and Pilon v1.18 [26–28]. Open reading frames (ORFs) in the genome were predicted using the GeneMark server (http://topaz.gatech.edu/GeneMark/genemarks.cgi) and RAST server (http://rast.nmpdr.org/rast.cgi). Each predicted ORF was annotated by performing a search against the NCBI non-redundant protein database and conserved domain database using BLASTp (https://blast.ncbi.nlm.nih.gov/Blast). Putative virulence factor and antibiotic resistance genes were screened against the Virulence Factor Database (VFDB, http://www.mgc.ac.cn/VFs/) [29] and Comprehensive Antibiotic Resistance Database (CARD, https://card.mcmaster.ca/analyze/rgi) [30].

### Codon usage and tRNA availability analysis

The codon usage was analyzed according to the online website (https://www.bioinformatics.org/sms2/index.html), accessed on 25 August 2023. Relative synonymous codon usage (RSCU) is the probability of the use of a particular codon in encoding codons corresponding to amino acid synonyms. If RSCU = 1.0, the codon was not biasis. If RSCU > 1.0, the codon was used preferentially. Conversely, RSCU < 1.0 suggested a negative bias in codon usage. Typically, codons with RSCU values > 1.6 were considered to be over-represented, whereas those < 0.6 were considered to be under-represented [31]. The tRNA availability was analyzed according to the online website with default settings (http://lowelab.ucsc.edu/tRNAscan-SE/), accessed on 21 August 2023.

### Whole genome sequence analysis of phage D6

The genome was visualized using the Proksee Server (https://proksee.ca/). The putative functions of these ORFs were annotated manually using the NCBI GenBank protein database (https://www.ncbi.nlm.nih.gov). A phylogenetic tree of terminal large subunit proteins was performed using neighbor-joining (NJ) method with MEGA 7. Bootstrap values were based on 1000 replicates. A comparative analysis of the phage genome sequences with its closest relative was conducted using Easyfig software [32]. The viral proteomic tree, including phage D6 and other closest relative phages, was constructed and analyzed using ViPTree (https://www.genome.jp/viptree/). The genome-wide average nucleotide identity (ANI) between phage D6 and related phages was calculated and plotted by Virus Intergenomic Distance Calculator (VIRIDIC) [33].

### In planta pathogenicity experiments

Foliar spraying (FS) and a root drenching (RD) methods [34] were used to further investigate the biocontrol efficacy of phage D6 against *Pst* DC3000 in tomato plants (*Solanum lycopersicum* var. *cerasiforme*). The tomato plants were cultured in a light incubator at 25 °C and 90% humidity with a photoperiod of 16 h. The plants at six-leaf stage with consistent growth were selected for the experiment. *Pst* DC3000 were cultured in KB medium containing 50 μg/mL rifampicin for 24 h at 28 °C and 180 rpm. Phage D6 was inoculated to *Pst* DC3000 and incubated for 12 h at 28 °C and 180 rpm, and the phage D6 preparation was obtained by filtration with a 0.22 μL filter membrane.

In FS and RD experiments, four treatment groups were set up: the control group (CK) treated with 10 mM magnesium chloride solution, the phage D6 treatment group was treated with 10 mL of phage D6 preparation (2.3 × 10^6^ PFU/mL, D6 group), the *Pst* treatment group was treated with 10 mL of *Pst* DC3000 (OD_600 =_ 0.2) culture, and the *Pst* + D6 treatment group was treated with phage D6 (2.3 × 10^6^ PFU/mL) for 24 h before being treated with *Pst* DC3000. Three tomato plants were used for each treatment group.

The in planta biocontrol effect of phage D6 was further evaluated by measuring the population of *Pst* DC3000 bacteria in plant leaves [34]. Briefly, the top, middle, and bottom leaves of the plants in each treatment group were collected after 14 d of treatment. Leaf discs of 1 cm diameter (1 cm^2^ /leaf) were cut off and a total of 9 cm^2^ of leaves were used (9 leaflets from 3 plants of per treatment group). The leaves were disinfected in 70% ethanol for 1 min and then rinsed with ddH_2_O for 30 s. Leaf homogenate was diluted tenfold and inoculated on KB plate (50 μg/mL rifampicin) and incubated at 28 °C for 48 h. Rifampicin-resistant mutants were screened and colony-forming units were counted.

### Statistical analysis

The resulting data were analyzed and visualized using SPSS 26. Significant differences were analyzed using one-way analysis of variance (ANOVA). All the experiments were performed in triplicate, and the results are expressed as the mean ± standard deviation (SD). A *p* value < 0.05 was considered statistically significant.

## Results

### Isolation and morphology of phage D6

**Fig. 1:**
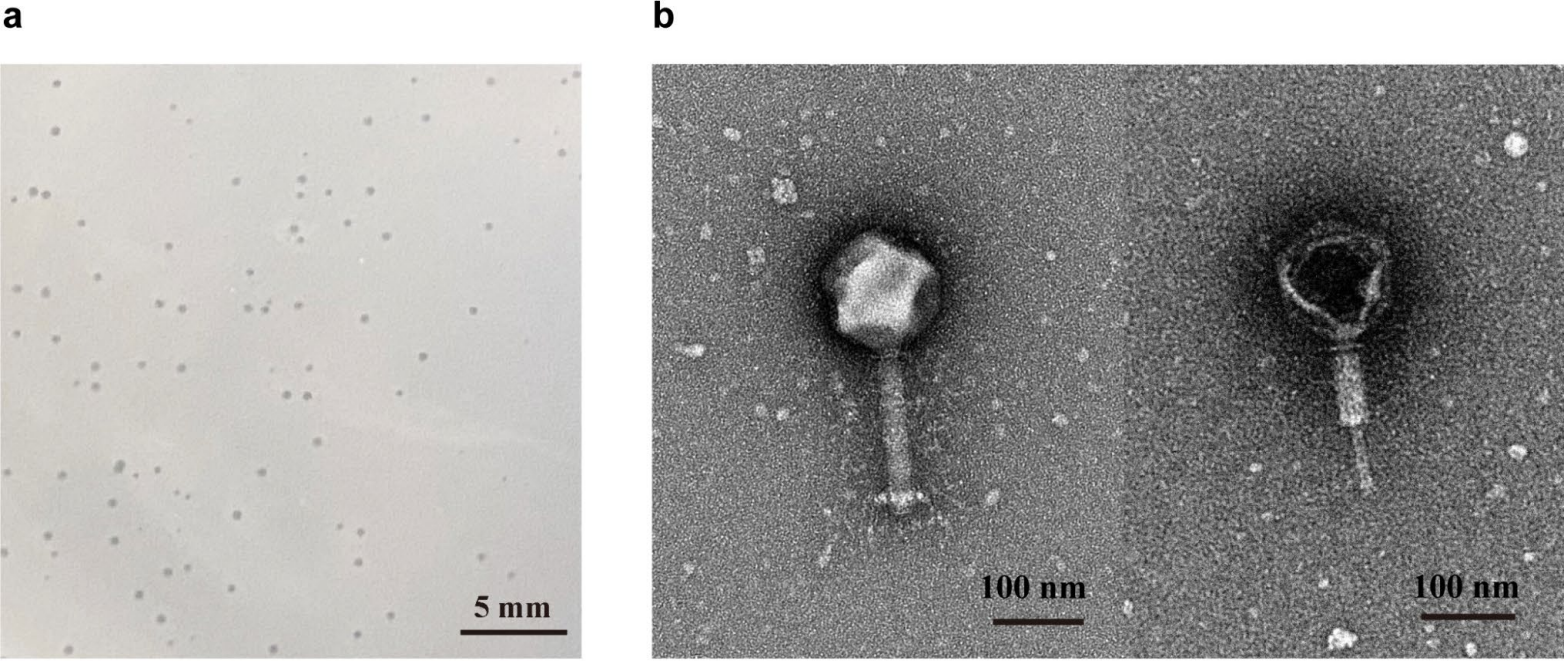
Morphology of phage D6. **a** Phage D6 plaques. Bar = 5 mm. **b** Morphology of phage D6 as revealed by transmission electron microscopy. Bar = 100 nm

A lytic phage effective against *Pst* DC3000, named D6, was isolated from cave sediments. Phage D6 formed translucent, round plaques with well-defined boundaries on double-layer agar plates (Fig. 1a), and had a supernatant titer of 1.0 × 10^8^ PFU/mL indicating completely lysis of host cells. Transmission electron microscopy analysis revealed that phage D6 had an icosahedral head (128.03 ± 1.57 nm in diameter) and a long, contractile tail (178.69 ± 0.56 nm in length) (Fig. 1b), consistent with the morphological characteristics of this type of phage. Based on these characteristics and the latest classification by the International Committee on Taxonomy of Viruses (ICTV), phage D6 exhibits the typical head and tail morphology of the class *Caudoviricetes* and can be morphologically classified as *Caudoviricetes*.

### Host range, EOP, and phage proteins analysis

The host range of phage D6 was tested using 11 strains of different *Pseudomonas* pp. isolated from karst cave. Phage D6 formed obvious spots on the lawn of six *Pseudomonas* strains (Supplementary Table S1), indicating that these strains were sensitive to the phage. The result of EOP assay showed that phage D6 did not form plaques on any of these six strains, indicating that phage D6 inhibited the growth of these strains but did not lyse [35]. The phage particle proteins of D6 were separated by SDS-PAGE, four bands with sizes of approximately 135, 75, 35, and 25 kDa were obtained (Supplementary Figure S1).

### Optimal MOI selection and one step growth curve assays

**Fig. 2:**
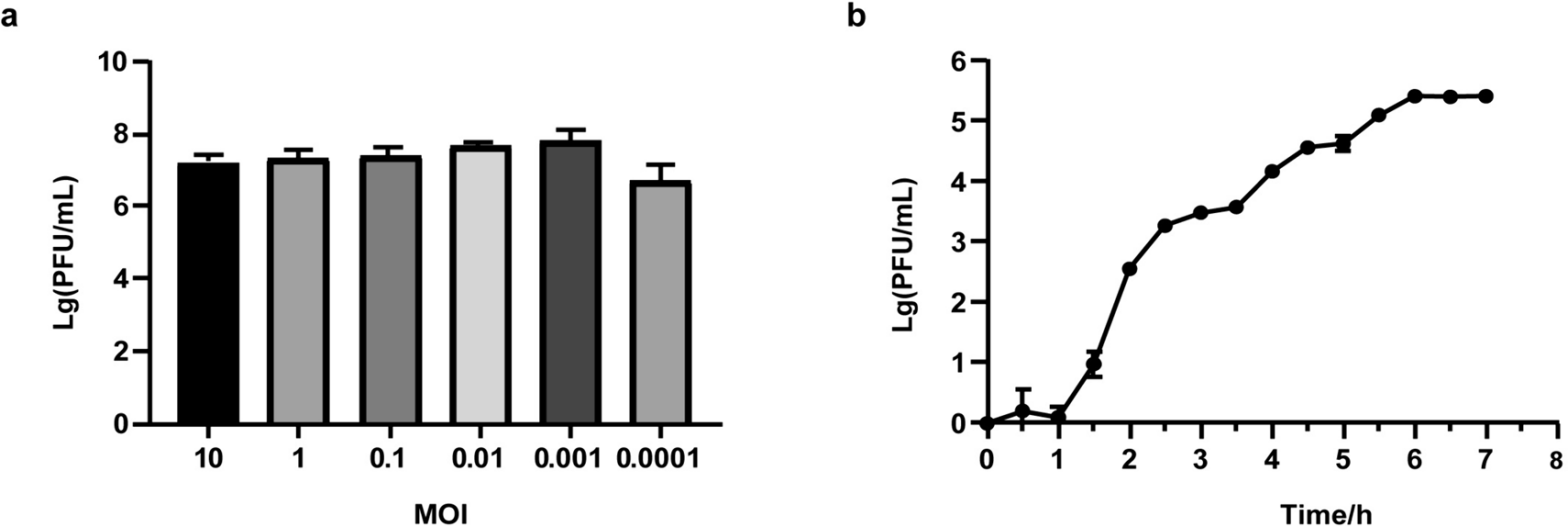
The optimal MOI (**a**) and one step growth curve (**b**) of phage D6. Data are presented as the average of triplicate experiments, and the error bars indicate the standard deviation

As shown in Fig. 2a, phage D6 mixed with *Pst* DC3000 at an MOI of 0.001 produced the highest phage titer (7.37 × 10^7^ PFU/mL). This indicates that an MOI of 0.001 is optimal for phage D6. The one step growth curve of phage D6 revealed an eclipse period of approximately 60 min and a 5 h rise phase (Fig. 2b). The average burst size of phage D6 was calculated to be around 16 PFU/cell.

### Thermal, pH, chloroform, and UV stability

**Fig. 3:**
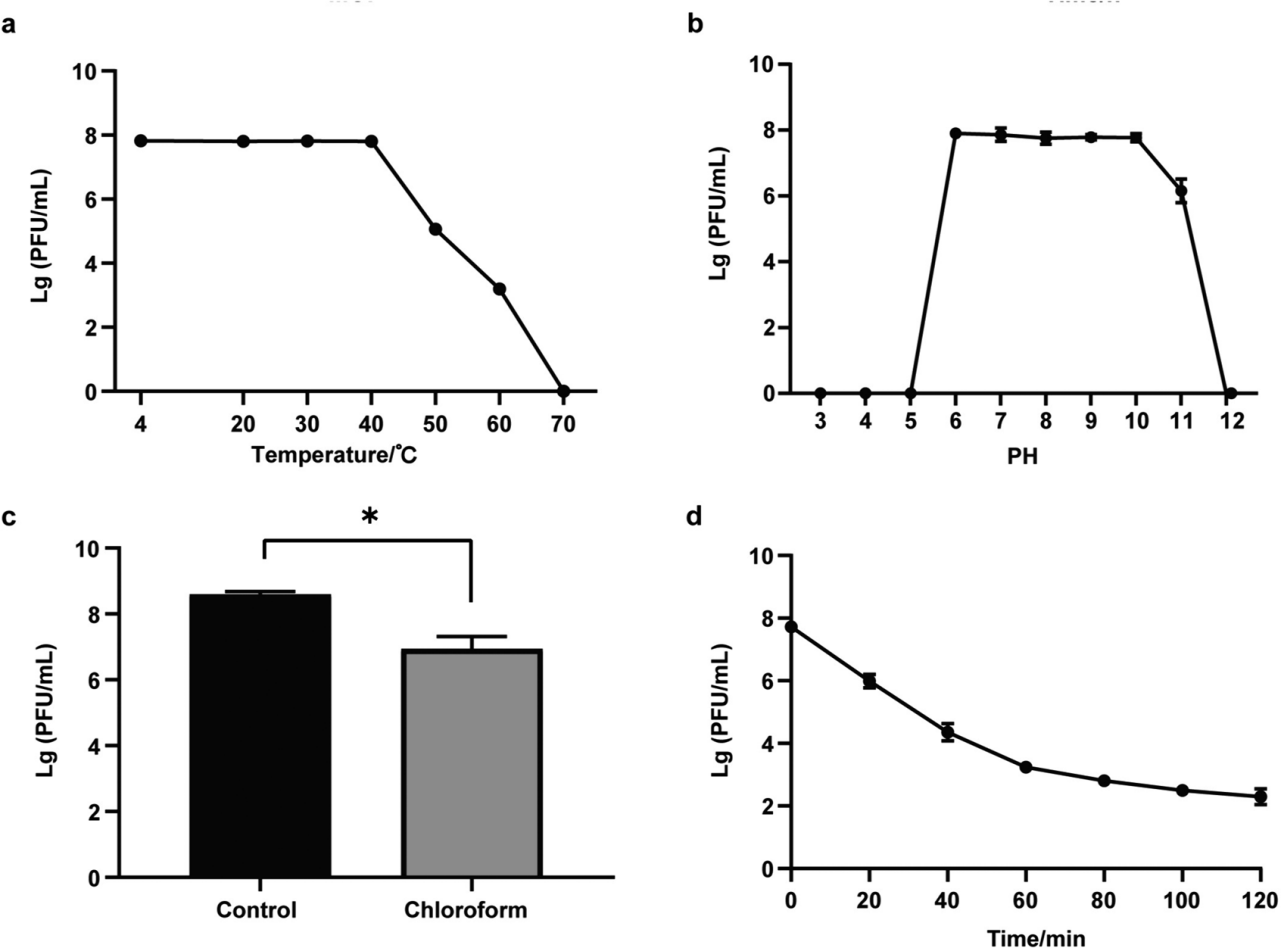
Thermal (**a**), pH (**b**), chloroform (**c**), and UV (**d**) stability of phage D6. Data are presented as the average of triplicate experiments, and the error bars indicate the standard deviation. *: significant difference (*p* < 0.05)

The titer of phage D6 remained stable (6.33 × 10^7^ − 6.63 × 10^7^ PFU/mL) at temperatures between 4 and 40 °C. The titer decreased as the temperature increased, dropping to 1.63 × 10^3^ PFU/mL at 60 °C and being inactivated at 70 °C (Fig. 3a). Phage D6 remained a high titer between pH 6 and pH 10 (10^8^ PFU/mL), but declined dramatically at pH 11, and was inactivated at pH ≤ 5 or ≥ 12 (Fig. 3b). These results indicate that the phage was tolerant to alkaline conditions. When mixed with 25% chloroform, the activity of phage D6 was decreased significantly compared with the blank control (*p* < 0.05) (Fig. 3c), indicating that phage D6 was sensitive to chloroform and suggesting that the phage particles may contain lipids [36]. The titer of phage D6 gradually decreased with increasing exposure to UV light, dropping to 220 PFU/mL after 2 h of irradiation (Fig. 3d).

### Adsorption assay

**Fig. 4:**
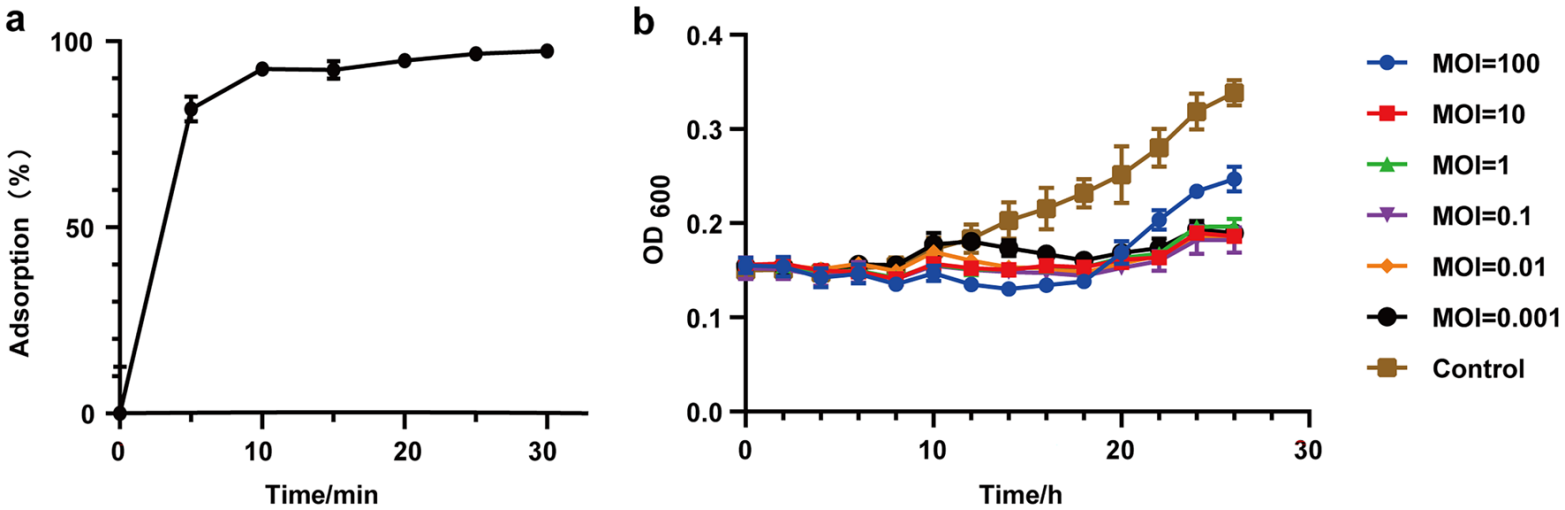
Adsorption rate (**a**) and bacteriostasis effect in vitro (**b**) of phage D6. Values are means from three independent experiments. Error bars indicate standard deviation

Phage D6 rapidly adsorbed to *Pst* DC3000 after co-culture, and the adsorption rate was 81.75% after only 5 min of incubation. The adsorption rate continued to increase slowly, reaching 92.54% after 10 min and 97.36% after 30 min (Fig. 4a).

### Bacteriostasis test in vitro

The bacteriostatic effect of phage D6 against *Pst* DC3000 was evaluated at different MOI (Fig. 4b). In general, phage D6 had a significant bacteriostatic effect on *Pst* DC3000 in an MOI-dependent manner, with higher MOIs resulting in a stronger inhibitory effects. When the MOI ranged from 0.001 to 10, phage D6 produced a long-term and stable bacteriostatic effect on the host bacterial population. However, when infected with a higher MOI (MOI = 100), the bacterial concentration began to increase again after 20 h. This suggests that even small amounts of phage D6 can significantly inhibit the proliferation of *Pst* DC3000.

### General genome characteristics

A total of 5,396,272 filtered reads were obtained and assembled into a complete genome of phage D6. The genome was 307,402 bp in length with a GC content of 48.43% (GenBank: OQ363659.1). Genome sequencing revealed that phage D6 had a linear double-stranded DNA. BLASTn analysis against the NCBI database showed that phage D6 had the highest genome similarity with *Pseudomonas* phage Psa21 (GenBank: NC-062581.1), with 81.20% nucleotide identity and over 41% genome coverage, suggesting that D6 is a relatively novel phage. The annotation results indicated that the phage D6 genome contains 410 ORFs. Of the 410 ORFs, 15 ORFs (3.66%) started with TTG, 21 ORFs (6.77%) started with GTG and the remaining ORFs (82.85%) started with ATG (Supplementary Table S2). The ORFs accounted for a total of 286,054 bp, and the gene density was 93.06%.

### Functional ORF analysis

**Fig. 5:**
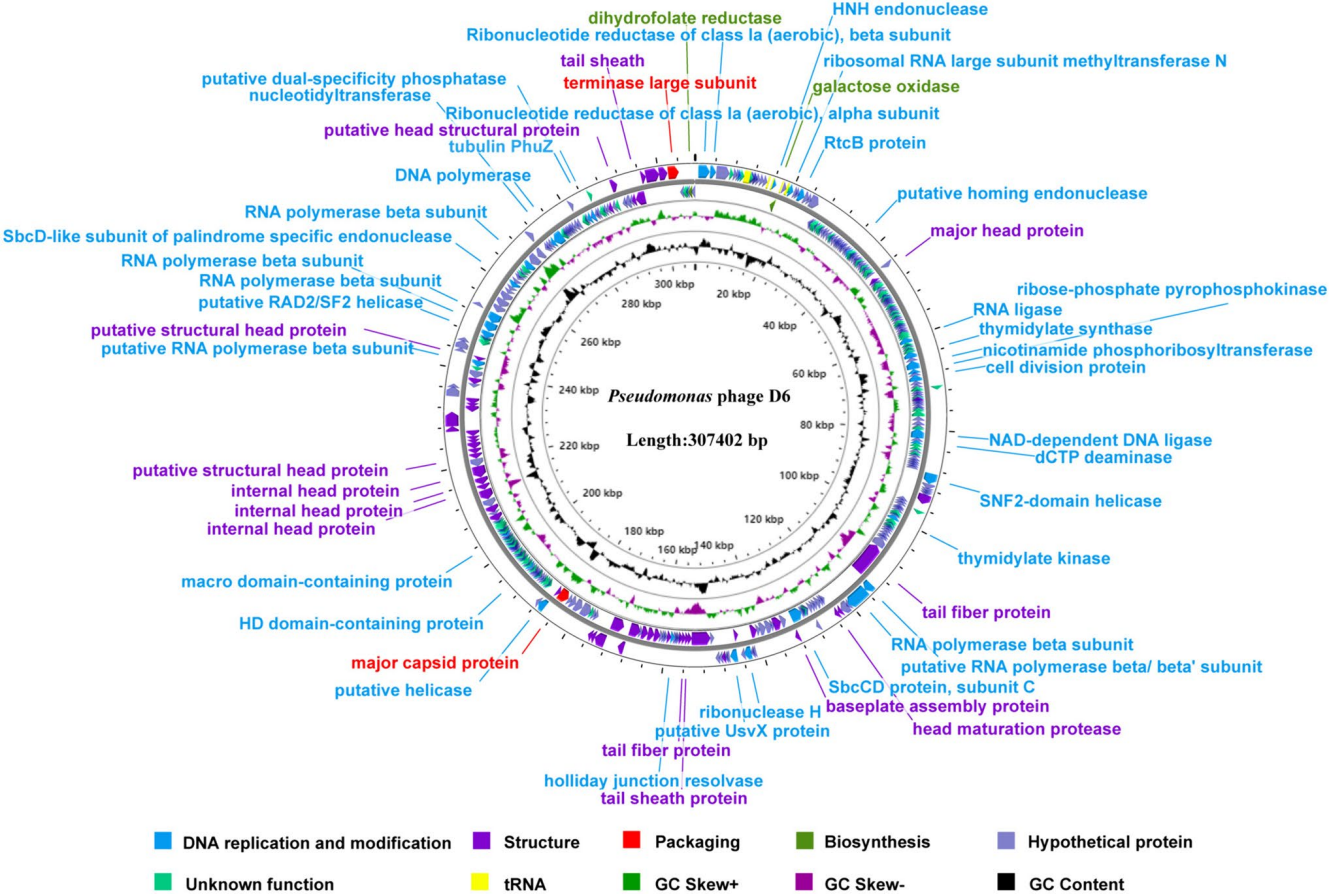
Genome map of phage D6. The genome of phage D6 was depicted in circular. The first and second circles from the outside to the inside are the ORFs on the positive and negative strands. The different colors represent different gene functions. The third circle represents the GC-Skew value. The fourth circle indicates the GC content

Of the 410 ORFs, 318 ORFs encoded known functional or hypothetical proteins, whereas the remaining 92 ORFs were not aligned to any known encoding proteins after BLASTp alignment. Based on the predicted functions of ORFs, the 92 ORFs encoding known functional proteins were divided into different groups (Fig. 5): structural proteins (54 ORFs), DNA replication and modification (34 ORFs), biosynthesis (2 ORFs), and DNA packaging (2 ORFs) (Supplementary Table S2).

Head and tail structural proteins are generally highly conserved in known phages [37]. In the structural proteins module, nine genes encode virion structural proteins such as the major capsid protein (ORF 251), tail fiber protein (ORF 181and ORF 224), tail sheath protein (ORF 223 and ORF 400), internal head protein (ORF 312, 313, and 314), and baseplate assembly protein (ORF 201). ORF 404 encodes a terminal large subunit protein of phage terminase, which is mainly involved in DNA translocation within the capsid as a part of a heterooligomer of the large subunit [38]. ORF 251 encodes the major capsid protein responsible for capsid formation, which can self-assembles into an icosahedral protocapsid with scaffold protein as a companion [6]. In the DNA replication and modification module, the predicted proteins encoded by ORF 182, ORF 183, ORF 333, ORF 344, ORF 345, ORF 360, and ORF 368 have primase and DNA/RNA polymerase activities. The DNA helicase proteins encoded by ORF 153, ORF 253, and ORF 343 unwind the double helix structure of DNA to facilitate replication [38]. ORF 25, ORF 107, and ORF 140 encode the ligases. ORF 18 and ORF 52 encode homing endonucleases, and ORF 18 belongs to the His-Asn-His (HNH) endonucleases family. ORF 211 encodes ribonuclease H, which is ubiquitously present in all prokaryotic cells. ORF 200 and ORF 352 encode nuclease SbcCD which can recognize and cleave hairpin structures generated during DNA replication. Finally, ORF118 and ORF384 encode proteins involved in cell division.

In the biosynthesis module, ORF 409 encodes dihydrofolate reductase while ORF 19 encodes galactose oxidase. Furthermore, no genes encoding virulence factors or antibiotic resistance were found in the genome of phage D6.

### Codon usage and tRNA availability prediction

To explore the relative synonymous codon usage preferences of phage D6 and the host, RSCU analysis was performed (Supplementary Table S3). Of 59 codons, 29 were the preferred codons for phage D6 and 28 were the preferred codons for the host. The preferred codon usage of phage D6 differs from that of the host, for example, the Alanine codon (GCA and GCT) were the preferred codons of phage D6, but had a negative bias on host codon usage. Furthermore, the over-represented codon for D6 had only one codon (CCA), whereas the host had two over-represented codon (GGC and CTG). These results suggested that codon usage had preferences between phage D6 and its hosts.

The tRNA availability analysis showed that phage D6 had 14 tRNAs corresponding to 12 amino acids, of which Asparaginate (Asn) and Leucine (Leu) amino acids corresponded to 2 tRNAs, respectively. The host had 2 tRNAs, corresponding to Serine (Ser) and Arginine (Arg) amino acids, respectively (Supplementary Table S4). Therefore, translation of the phage D6 gene may not be entirely dependent on the tRNA repertoire provided by the host.

### Whole genome sequence analysis and phylogenetic analysis

**Fig. 6:**
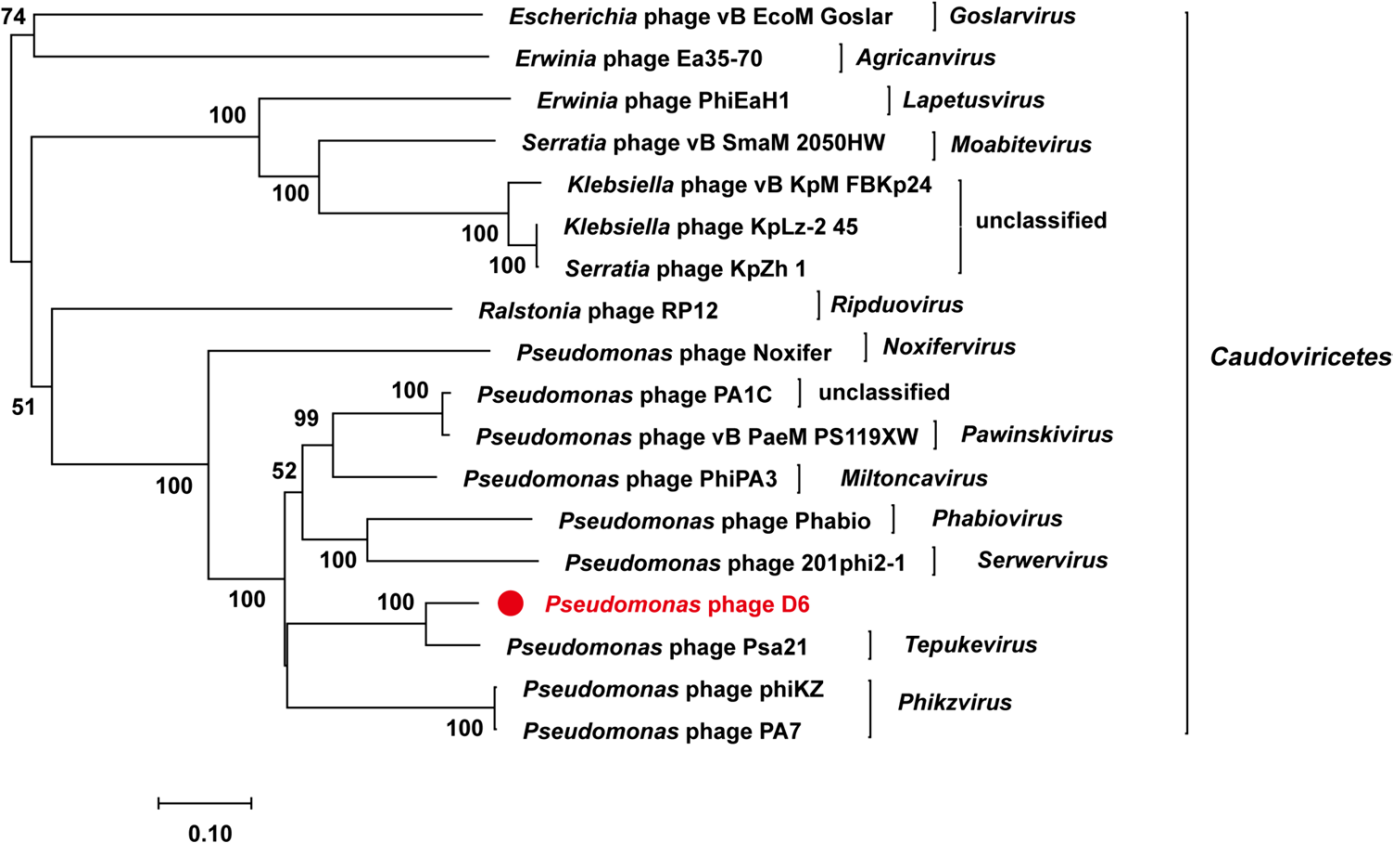
Neighbor-joining phylogenetic tree was constructed based on terminase large subunit. The bootstrap values are shown at the nodes. The red star represents phage D6 isolated in this study. The reference sequences were collected from the NCBI database. The tree was constructed based on the MUSCLE alignment of MEGA 7.0. The bootstrap values were based on 1000 replicates

To analyze the evolutionary relationship between phage D6 and other *Caudoviricetes* phages, a phylogenetic tree was constructed using the neighbor-joining (NJ) method based on the amino acid sequence of the relatively conserved phage terminal large subunit. The phylogenetic tree showed that phages D6 and Psa21 clustered together on a single branch, distinct from other *Pseudomonas* phages. This suggests that the *Pseudomonas* phage may represent a new and distinct group with the *Caudoviricetes* class (Fig. 6).

**Fig. 7:**
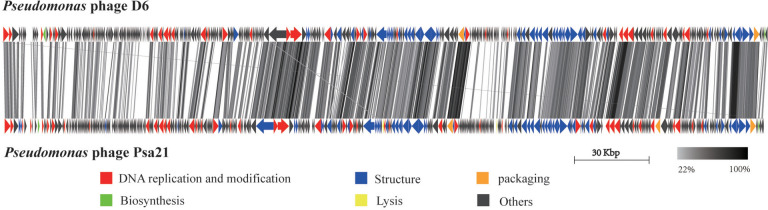
Pairwise comparisons of the whole genomes of *Pseudomonas* phage D6 and phage Psa21 was visualized using Easyfig. The arrows are the encoded proteins and the gray line connecting the two represents the similarity between the two, with darker shades of gray representing higher similarity

A BLASTn analysis of the genome revealed that phage D6 was most closely related to Psa21, a *Pseudomonas* phage. Further comparative genomic analysis of these two phages demonstrated that more than half of the proteins they encoded were hypothetical proteins. Of these, 310 proteins (75.61%) encoded by D6 were identical to those encoded by Psa21, with similarity ranging from 22 to 100% (Fig. 7). Moreover, the genome maps showed significant differences between the complete genome sequences of phage D6 and Psa21 (Fig. 7).

Taken together, the phylogenetic tree analysis and comparative genomic analysis suggest that phage D6, which infects *Pst* DC3000, is a novel unclassified phage within the *Caudoviricetes* class.

**Fig. 8:**
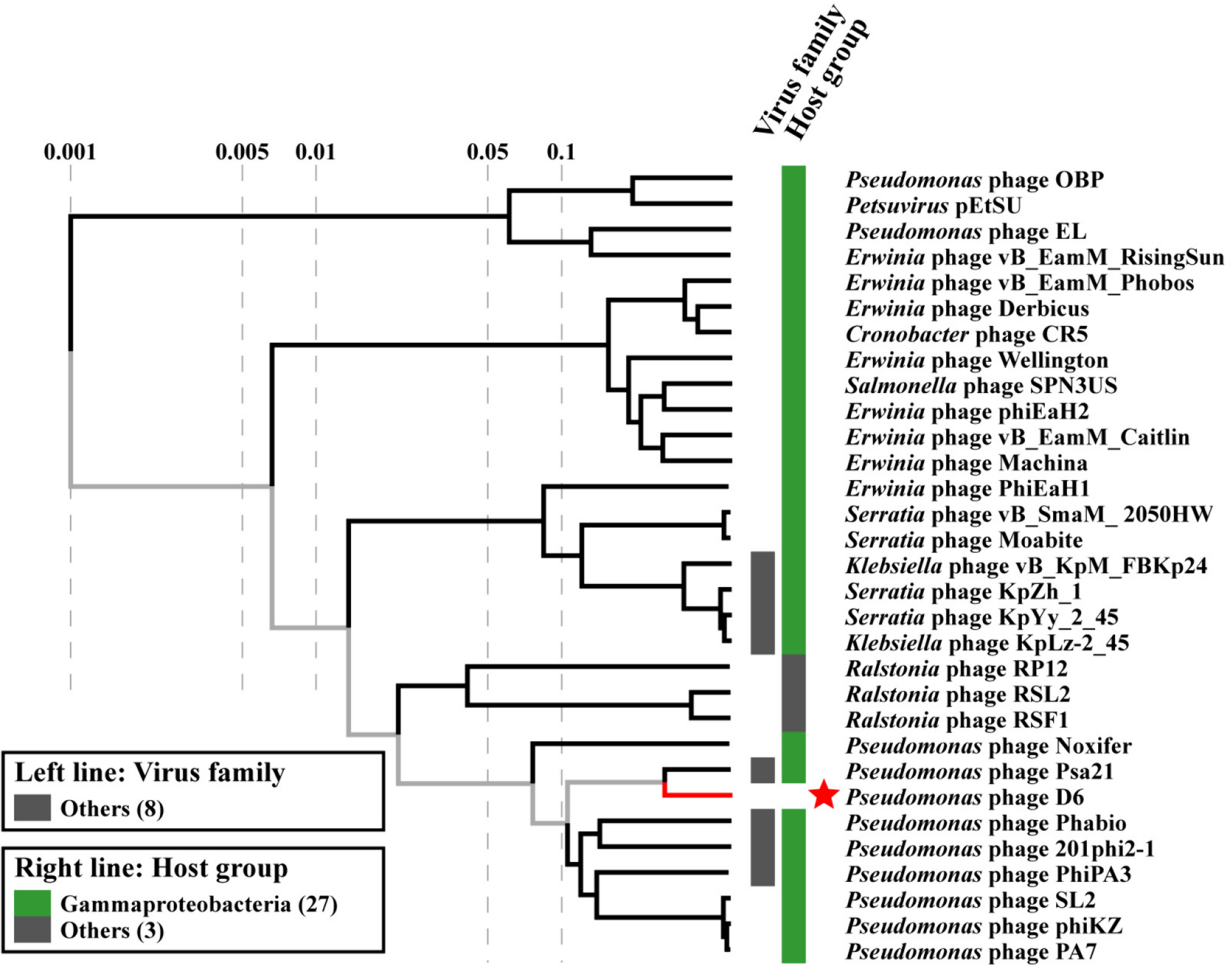
Comparative proteomic phylogenetic analysis of phage D6 and similar phages performed using VipTree. The red star represents *Pseudomonas* phage D6 isolated in this study. The phylogenetic branches marked in red are represented as phage sequence not found in the Virus-Host Database. Additional genomes were downloaded from the NCBI GenBank database and manually added to the analysis. Left line represents virus family, right line represents host group

A comparative proteomic phylogenetic analysis using ViPTree software showed that the genomes of phage D6 and phage Psa21 formed a distinct branch. Phage D6 was most closely related to *Pseudomonas* phage Psa21, but was distinguished from other phages in the VipTree database, indicating that it may represent a novel phage within the *Caudoviricetes* class (Fig. 8).

**Fig. 9:**
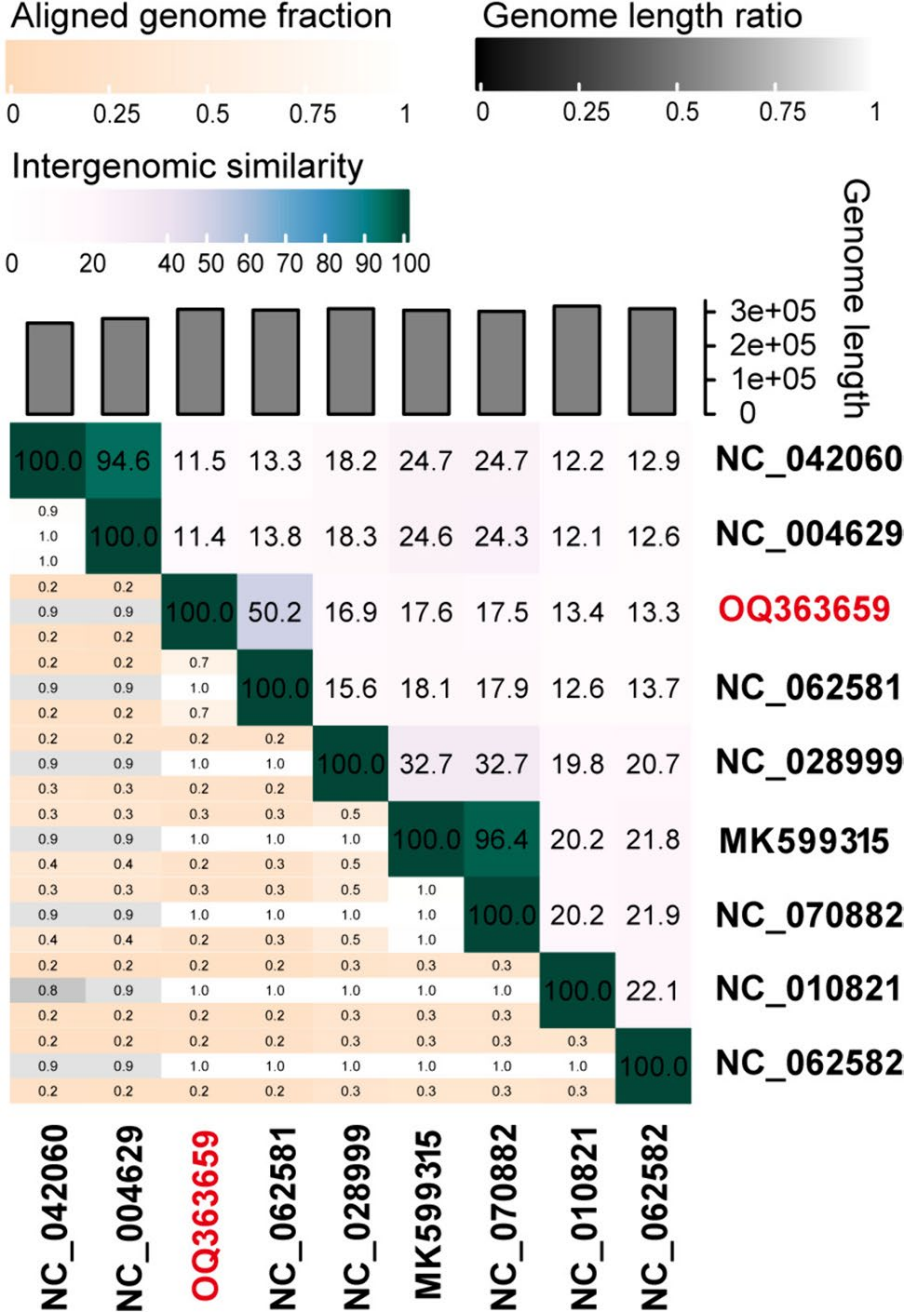
Heatmap of genome sequence similarity (right) and alignment metrics (left) between phage D6 and its eight most closely related phages generated by VIRIDIC. The red number represents phage D6 isolated in this study

To further clarify the classification of phage D6, we used VIRIDIC software to caculate the matrix of intergenomic similarities between D6 (OQ363659) and eight of the most similar phage genomes (*Pseudomonas* phage phiKZ (NC_004629), *Pseudomonas* phage PA7 (NC_042060), *Pseudomonas* phage Psa21 (NC_062581), *Pseudomonas* phage PhiPA3 (NC_028999), *Pseudomonas* phage PA1C (MK599315), *Pseudomonas* phage vB_PaeM_PS119XW (NC_070882), *Pseudomonas* phage 201phi2-1 (NC_010821), and *Pseudomonas* phage Phabio (NC_062582)). The results showed that the highest similarity value of 50.2% was between *Pseudomonas* phage D6 and Psa21 (Fig. 9). The pairwise similarity of the genomes was lower than the cut-off value of 70% nucleotide identity across the whole genome length established by the ICTV Bacterial Virus Subcommittee for creating phage genera [39]. Thus, phage D6 should be considered a member of a novel genus within the *Caudoviricetes* class.

### Evaluation of plant protective activity of D6 against *Pst* DC3000 in planta

**Fig. 10:**
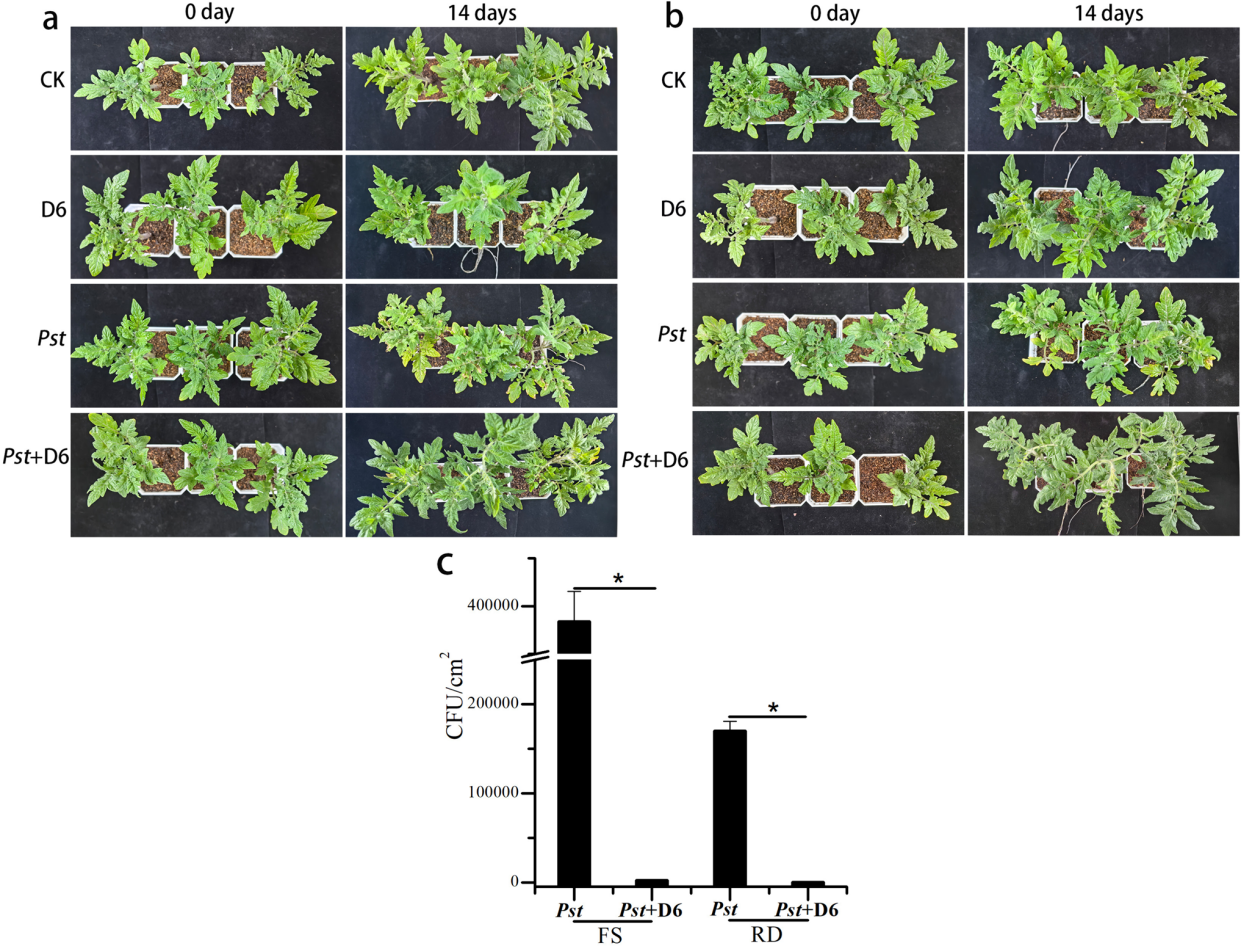
Biocontrol evaluation of phage D6 against *Pst* DC3000 in planta. After 24 h of inoculation with 10 mL of phage D6 (2.3 × 10^6^ PFU/mL) by the FS method (**a**) and RD method (**b**), tomato plants were infected with 10 mL of *Pst* DC3000 culutre (*Pst* + D6 group). The leaf symptoms were observed. The tomato plants were treated with phage D6, *Pst* DC3000 (OD_600_ = 0.2), or magnesium chloride solution (10 mM) (CK group) were used as controls. (**c**) After 14 d of treatment, the top, middle, and bottom leaves of the plants were collected to detect the amounts of *Pst* DC3000 bacteria. The experiments were performed in triplicates and the data are shown as the mean ± SD. *: significant difference (*p* < 0.05)

Tomato plants were inoculated with phage D6 24 h prior to *Pst* DC3000 infection using the FS method (Fig. 10a) and RD method (Fig. 10b), and the quantity of *Pst* DC3000 in the leaves was measured (Fig. 10c) in order to investigate the plant protective efficiency of phage D6 against *Pst* DC3000 in vivo*.* The findings demonstrated that neither bacterial leaf speck disease nor bacteria were found in the CK or D6 treatment group following a 14 days treatment. The tomato plants that were infected with *Pst* DC3000 showed obvious bacterial leaf speck disease, leaf withered, wilting symptoms, and leaf yellowing. However, the leaf symptoms such as bacterial wilting and yellowing were alleviated in the *Pst* + D6 group inoculated with phage D6 (Fig. 10a, b). Moreover, the amount of *Pst* DC3000 in tomato leaves was significantly decreased after inoculation with phage D6 (Fig. 10c). The *Pst* DC3000 population in the *Pst* + D6 treatment group was significantly reduce by 150-fold and 263-fold in the FS and RD treatment, respectively, compared with the *Pst* treatment group (*p* < 0.05). These results indicate that D6 inoculation using both FS and RD methods could effectively control the amount of *Pst* DC3000 in tomato plants, thereby reducing the damage caused by *Pst* DC3000 infection.

## Discussion

In this study, a novel phage named D6, which infects the plant pathogen *P. syringae* pv. *tomato,* was isolated from sediment in a karst cave. The morphology and genome of phage D6 were characterized, and a combination of whole genome sequence analysis, evolutionary phylogenetic analysis, comparative genomic analysis, and comparative proteomic phylogenetic analysis indicated that it is a novel unclassified *Pseudomonas* phage within the *Caudoviricetes* class.

The genome length of phage D6 is 307,402 bp, suggesting that it belongs to be the group of jumbo phage [40]. With its large physical genome size, phage D6 shares similar characteristics with closely related phages Psa21 and PhiPA3 [41–43]. It has been proposed that jumbo phages with large genomes have more genomic space to undergo higher rates of genetic evolution in comparison with their smaller size and more genomically compact [40]. This is hypothesized to be an adaptive mechanism for phage evolution to evade bacterial defense systems [44–46]. The large number of transcribed and translated genes in the virus reduces its dependence on the host, allowing jumbo phages to develop infections that are highly independent of host molecular mechanisms [47, 48]. Phage D6 forms small translucent plaques on double-layer agar plates, which may be limited by the influence of the agar medium on spreading [49].

The ability to survive under different environmental conditions is crucial for the use of phages in biocontrol. Phage D6 was sensitive to several isolates, of *pseudomonas* spp. But was only bacteriostatic and unable lyse the test host. Phage D6 can maintain a stable titer at temperature ranging from 4 to 40 °C and pH from 6 to 10, which is conducive to its use in weakly acidic to alkaline natural environments. Additionally, phage D6 has a lower optimal MOI (0.001), a fast adsorption rate, a short eclipse period (approximately 60 min), and a short rise phase (5 h). In vitro bacteriostatic experiments showed that low MOI (0.001–10) produced long-term stable bacteriostatic effect on the bacterial population, with *Pst* DC3000 replication significantly inhibited by small amounts of phage D6. The lower titer requirement of phage D6 makes it more suitable for large-scale production and field application.

Among the structural proteins, 54 ORFs were predicted that encode virion structural proteins such as the major capsid protein (ORF 251), tail protein (4 ORFs), and head protein (8 ORFs). Capsid proteins are essential structural components of the virion that protect the viral genome from harsh external environment [50]. The phage tail is the primary site for recognizing and adsorbing host bacteria and is key to expanding the phage’s host range [51]. In the DNA replication, transcription, and translation module, 34 ORFs were predicted to encode replication-related enzymes such as RNA polymerase (6 ORFs), homing endonucleases (2 ORFs), and HNH endonucleases (ORF 18). RNA polymerase is the central enzyme in gene expression, transcribing DNA into RNA.

Homing endonucleases are a unique class of site-specific DNA endonucleases present in many bacterial and phage genomes that facilitate genetic exchange and horizontal transfer of their own genes and local genetic environment [52, 53]. HNH endonucleases are a large group of proteins identified in various organisms, including bacteriophages and bacteria. They are associated with DNA packaging and have been described as a cofactor associated with terminases for many phages [54, 55]. Additionally, phage D6 also encodes a variety of enzymes involved in replication, repair, and recombination, such as tubulin PhuZ (ORF 384) and UvsX protein (ORF 214). Tubulins PhuZ belong to a bacteriophage tubulin family that has been shown to assemble dynamic filaments that locate phage DNA in the center of the cell during the phage reproduction cycle, contributing to efficient phage reproduction [8, 56]. The UvsX protein encoded by ORF214, similar to the *E. coli* RecA protein, is a DNA-dependent ATP enzyme that catalyzes the pairing of cognate DNA molecules [57]. Furthermore, 14 tRNA genes were identified, suggesting that phage D6 may not be highly dependent on the host translation machinery.

Based on an NCBI database search, phage D6 had the highest genome similarity with *Pseudomonas* phage Psa21, with a nucleotide similarity of 81.20% and a genome coverage of over 41%, suggesting that D6 is a relatively new phage. Phages D6 and Psa21 are closely related. However, in earlier studies, phage Psa21 was shown to be a member of the *Myoviridae* family. Nonetheless, according to the ICTV Master Species List 2022 (https://talk.ictvonline.org, accessed on 9 January 2024) and a BLASTn search in the NCBI database, we propose that phage D6 is an unclassified novel *Pst* phage within the *Caudoviricetes* class.

Inoculation with phage D6 by the FS or RD method significantly reduced the population of *Pst* DC3000 in the leaflets surface and alleviated the *Pst* symptoms in tomato plants, indicating that phage D6 can be used as a prophylactic treatment for *Pst* DC3000. Previously results have been reported the biological control of *Pst* DC3000 by phages in planta [2, 34]. The biological properties of phage D6 allow us to propose it a potential biocontrol agent for the prevention of *Pst*-associated bacterioses.

## Conclusion

In this study, a novel *Pseudomonas* phage named D6 was isolated from a karst cave. Phage D6 exhibits a wide range of pH from weakly acidic to alkaline as well as room temperature environment stability. Genome sequencing analysis revealed that phage D6 had a linear double-stranded DNA (307,402 bp) with a G + C content of 48.43% and contained 410 ORFs and 14 tRNAs. NCBI alignment results showed that phage D6 had the highest genome similarity with *Pseudomonas* phage Psa21 (NC_062581), with a nucleotide similarity of 81.20% and a genome coverage of over 41%. Phylogenetic analysis using terminase large subunit protein sequences and viral proteomic tree revealed that phage D6 was highly related to phages Psa21, and that D6 and Psa21 clustered into an unclassified monophyletic virus cluster, D6 belongs to a novel representative of this clade within the *Caudoviricetes* class. Thus, based on genome-wide analysis and phylogenetic analysis, phage D6 could represent a novel *Pseudomonas* phage assigned to the unclassified *Caudoviricetes* according to the classification of viruses by ICTV. Furthermore, phage D6 can be used as a potential biocontrol agent for the prevention of *Pst*-associated bacterioses.

### Supplementary Information

Below is the link to the electronic supplementary material.

Supplementary file1 (DOCX 45 kb)

## Data Availability

The data presented in this study are available from the corresponding author on reasonable request.

## References

[1] Cheng SS, Ku YS, Cheung MY, Lam HM (2022). Identification of stably expressed reference genes for expression studies in *Arabidopsis*
*thaliana* using mass spectrometry-based label-free quantification. Front Plant Sci.

[2] Korniienko N, Kharina A, Zrelovs N, Jindřichová B, Moravec T, Budzanivska I (2022). Isolation and characterization of two lytic phages efficient against phytopathogenic bacteria from *Pseudomonas* and *Xanthomonas* Genera. Front Microbiol.

[3] Altimira F, Yáñez C, Bravo G, González M, Rojas LA, Seeger M (2012). Characterization of copper-resistant bacteria and bacterial communities from copper-polluted agricultural soils of central Chile. BMC Microbiol.

[4] Pinheiro LAM, Pereira C, Frazão C, Balcão VM, Almeida A (2019). Efficiency of phage φ6 for biocontrol of *Pseudomonas*
*syringae* pv. syringae: an in vitro preliminary study. Microorganisms.

[5] Xin XF, Kvitko B, He SY (2018). *Pseudomonas syringae*: what it takes to be a pathogen. Nat Rev Microbiol.

[6] Liu Y, Liu M, Hu R, Bai J, He X, Jin Y (2021). Isolation of the novel phage PHB09 and its potential use against the plant pathogen *Pseudomonas*
*syringae* pv. actinidiae. Viruses.

[7] Danis-Wlodarczyk K, Vandenheuvel D, Jang HB, Briers Y, Olszak T, Arabski M (2016). A proposed integrated approach for the preclinical evaluation of phage therapy in *Pseudomonas* infections. Sci Rep.

[8] Kraemer James A, Erb Marcella L, Waddling Christopher A, Montabana Elizabeth A, Zehr Elena A, Wang H (2012). A phage tubulin assembles dynamic filaments by an atypical mechanism to center viral DNA within the host cell. Cell.

[9] Kauppinen A, Siponen S, Pitkänen T, Holmfeldt K, Pursiainen A, Torvinen E (2021). Phage biocontrol of *Pseudomonas aeruginosa* in water. Viruses.

[10] Akremi I, Merabishvili M, Jlidi M, Haj Brahim A, Ben Ali M, Karoui A (2022). Isolation and characterization of lytic *Pseudomonas aeruginosa* bacteriophages isolated from sewage samples from tunisia. Viruses.

[11] Evseev P, Sykilinda N, Gorshkova A, Kurochkina L, Ziganshin R, Drucker V (2020). *Pseudomonas* phage PaBG-A jumbo member of an old parasite family. Viruses.

[12] Rathor N, Thakur CK, Das BK, Chaudhry R (2022). An insight into the therapeutic potential of a novel lytic *Pseudomonas* phage isolated from the river Ganga. J Appl Microbiol.

[13] Patpatia S, Yilmaz O, Ylänne M, Kiljunen S (2021). Isolation and genomic analysis of the Phage vB_PaeP_fHoPae04 infecting *Pseudomonas aeruginosa*. Microbiol Resour Announc.

[14] Song YR, Vu NT, Park J, Hwang IS, Jeong HJ, Cho YS (2021). Phage PPPL-1, a new biological agent to control bacterial canker caused by *Pseudomonas*
*syringae* pv. actinidiae in Kiwifruit. Antibiotics (Basel, Switzerland).

[15] Wright A, Hawkins CH, Anggård EE, Harper DR (2009). A controlled clinical trial of a therapeutic bacteriophage preparation in chronic otitis due to antibiotic-resistant *Pseudomonas*
*aeruginosa*; a preliminary report of efficacy. Clin Otolaryngol.

[16] Cafora M, Deflorian G, Forti F, Ferrari L, Binelli G, Briani F (2019). Phage therapy against *Pseudomonas aeruginosa* infections in a cystic fibrosis zebrafish model. Sci Rep.

[17] Jault P, Leclerc T, Jennes S, Pirnay JP, Que YA, Resch G (2019). Efficacy and tolerability of a cocktail of bacteriophages to treat burn wounds infected by *Pseudomonas aeruginosa* (PhagoBurn): a randomised, controlled, double-blind phase 1/2 trial. Lancet Infect Dis.

[18] Dong Y, Gao J, Wu Q, Ai Y, Huang Y, Wei W (2020). Co-occurrence pattern and function prediction of bacterial community in Karst cave. BMC Microbiol.

[19] Zhang Q, Xing S, Sun Q, Pei G, Cheng S, Liu Y (2017). Characterization and complete genome sequence analysis of a novel virulent *Siphoviridae* phage against *Staphylococcus aureus* isolated from bovine mastitis in Xinjiang China. Virus Genes.

[20] Yang M, Chen H, Huang Q, Xie Z, Liu Z, Zhang J (2022). Characterization of the novel Phage vB_VpaP_FE11 and its potential role in controlling *Vibrio parahaemolyticus* biofilms. Viruses.

[21] Lee SY, Thapa Magar R, Kim HJ, Choi K, Lee SW (2021). Complete genome sequence of a novel bacteriophage RpY1 infecting *Ralstonia solanacearum* strains. Current Microbiol.

[22] Fujiki J, Yoshida SI, Nakamura T, Nakamura K, Amano Y, Nishida K (2021). Novel virulent bacteriophage ΦSG005, which infects *Streptococcus gordonii*, forms a distinct clade among *Streptococcus* viruses. Viruses.

[23] Schubert M, Lindgreen S, Orlando L (2016). AdapterRemoval v2: rapid adapter trimming, identification, and read merging. BMC Res Notes.

[24] Coil D, Jospin G, Darling AE (2015). A5-miseq: an updated pipeline to assemble microbial genomes from Illumina MiSeq data. Bioinformatics.

[25] Bankevich A, Nurk S, Antipov D, Gurevich AA, Dvorkin M, Kulikov AS (2012). SPAdes: a new genome assembly algorithm and its applications to single-cell sequencing. J Comput Biol.

[26] Altschul SF, Gish W, Miller W, Myers EW, Lipman DJ (1990). Basic local alignment search tool. J Mol Biol.

[27] Kurtz S, Phillippy A, Delcher AL, Smoot M, Shumway M, Antonescu C (2004). Versatile and open software for comparing large genomes. Genome Biol.

[28] Walker BJ, Abeel T, Shea T, Priest M, Abouelliel A, Sakthikumar S (2014). Pilon: an integrated tool for comprehensive microbial variant detection and genome assembly improvement. PLoS ONE.

[29] Liu B, Zheng D, Jin Q, Chen L, Yang J (2019). VFDB 2019: a comparative pathogenomic platform with an interactive web interface. Nucleic Acids Res.

[30] Alcock BP, Huynh W, Chalil R, Smith KW, Raphenya AR, Wlodarski MA (2023). CARD 2023: expanded curation, support for machine learning, and resistome prediction at the comprehensive antibiotic resistance database. Nucleic Acids Res.

[31] Zhou J, Wang X, Zhou Z, Wang S (2023). Insights into the evolution and host adaptation of the monkeypox virus from a codon usage perspective: focus on the ongoing 2022 outbreak. Int J Mol Sci.

[32] Sullivan MJ, Petty NK, Beatson SA (2011). Easyfig: a genome comparison visualizer. Bioinformatics.

[33] Moraru C, Varsani A, Kropinski AM (2020). VIRIDIC-A novel tool to calculate the intergenomic similarities of prokaryote-infecting viruses. Viruses.

[34] Skliros D, Papazoglou P, Gkizi D, Paraskevopoulou E, Katharios P, Goumas DE (2023). In planta interactions of a novel bacteriophage against *Pseudomonas*
*syringae* pv. tomato. App Microbiol Biotechnol.

[35] Vashisth M, Jaglan AB, Yashveer S, Sharma P, Bardajatya P, Virmani N (2023). Development and evaluation of bacteriophage cocktail to eradicate biofilms formed by an extensively drug-Resistant (XDR) *Pseudomonas aeruginosa*. Viruses.

[36] Kauffman KM, Hussain FA, Yang J, Arevalo P, Brown JM, Chang WK (2018). A major lineage of non-tailed dsDNA viruses as unrecognized killers of marine bacteria. Nature.

[37] Zhang W, Zhang R, Hu Y, Liu Y, Wang L, An X (2021). Biological characteristics and genomic analysis of a *Stenotrophomonas maltophilia* phage vB_SmaS_BUCT548. Virus Genes.

[38] Xiang Y, Li W, Song F, Yang X, Zhou J, Yu H (2020). Biological characteristics and whole-genome analysis of the *Enterococcus faecalis* phage PEf771. Canadian J Microbiol.

[39] Turner D, Kropinski AM, Adriaenssens EM (2021). A roadmap for genome-based phage taxonomy. Viruses.

[40] Yuan Y, Gao M (2017). Jumbo bacteriophages: an overview. Front Microbiol.

[41] Wojtus JK, Frampton RA, Warring S, Hendrickson H, Fineran PC (2019). Genome sequence of a jumbo bacteriophage that infects the kiwifruit phytopathogen *Pseudomonas*
*syringae* pv. actinidiae. Microbiol Resour Announc.

[42] Chaikeeratisak V, Nguyen K, Egan ME, Erb ML, Vavilina A, Pogliano J (2017). The phage nucleus and tubulin spindle are conserved among large *Pseudomonas* phages. Cell Rep.

[43] Monson RE, Foulds IJ, Foweraker JE, Welch M, Salmond GPCJM (2011). The *Pseudomonas aeruginosa* generalized transducing phage phiPA3 is a new member of the phiKZ-like group of 'jumbo' phages, and infects model laboratory strains and clinical isolates from cystic fibrosis patients. Microbiology.

[44] Chaikeeratisak V, Nguyen K, Khanna K, Brilot AF, Erb ML, Coker JK (2017). Assembly of a nucleus-like structure during viral replication in bacteria. Science.

[45] Ofir G, Sorek R (2018). Contemporary phage biology: from classic models to new insights. Cell.

[46] Hendrix RW (2009). Jumbo bacteriophages. Curr Topics Microbiol Immunol.

[47] Ceyssens PJ, Minakhin L, Van den Bossche A, Yakunina M, Klimuk E, Blasdel B (2014). Development of giant bacteriophage ϕKZ is independent of the host transcription apparatus. J Virol.

[48] Lavysh D, Sokolova M, Minakhin L, Yakunina M, Artamonova T, Kozyavkin S (2016). The genome of AR9, a giant transducing *Bacillus* phage encoding two multisubunit RNA polymerases. Virology.

[49] Serwer P, Hayes SJ, Thomas JA, Hardies SCJVJ (2007). Propagating the missing bacteriophages: a large bacteriophage in a new class. Virol J.

[50] Hungaro HM, Vidigal PMP, Nascimento EC, da Costa G, Oliveira F, Gontijo MTP, Lopez MES (2022). Genomic characterisation of UFJF_PfDIW6: a novel lytic *Pseudomonas fluorescens* phage with potential for biocontrol in the dairy industry. Viruses.

[51] Leiman PG, Shneider MM (2012). Contractile tail machines of bacteriophages. Adv Exp Med Biol.

[52] Belfort M, Bonocora RP (2014). Homing endonucleases: from genetic anomalies to programmable genomic clippers. Methods Mol Biol.

[53] Friedrich NC, Torrents E, Gibb EA, Sahlin M, Sjöberg BM, Edgell DR (2007). Insertion of a homing endonuclease creates a genes-in-pieces ribonucleotide reductase that retains function. Proc Nat Acad Sci USA.

[54] Quiles-Puchalt N, Carpena N, Alonso JC, Novick RP, Marina A, Penadés JR (2014). Staphylococcal pathogenicity island DNA packaging system involving cos-site packaging and phage-encoded HNH endonucleases. Proc Nat Acad Sci USA.

[55] Kala S, Cumby N, Sadowski PD, Hyder BZ, Kanelis V, Davidson AR (2014). HNH proteins are a widespread component of phage DNA packaging machines. Proc Nat Acad Sci USA.

[56] Erb ML, Kraemer JA, Coker JK, Chaikeeratisak V, Nonejuie P, Agard DA (2014). A bacteriophage tubulin harnesses dynamic instability to center DNA in infected cells. Elife.

[57] Harris LD, Griffith JD (1988). Formation of D loops by the UvsX protein of T4 bacteriophage: a comparison of the reaction catalyzed in the presence or absence of gene 32 protein. Biochemistry.

