# Supplementary material for: Characteristics and whole-genome analysis of a novel Pseudomonas syringae pv. tomato bacteriophage D6 isolated from a karst cave
Source: Virus Genes. 2024 Apr 9;60(3):295–308. doi: 10.1007/s11262-024-02064-9 (PMC11139720; doi:10.1007/s11262-024-02064-9)
Supplement: Supplementary file 1 — Supplementary file1 (DOCX 45 kb) [file 11262_2024_2064_MOESM1_ESM.docx]

# Characteristics and whole-genome analysis of a novel *Pseudomonas syringae* pv. *tomato* bacteriophage D6 isolated from a karst cave

**Author names**

Qingshan Wu^1#^, Ni An^1#^, Zheng Fang^1^, Shixia Li^1^, Lan Xiang^2^, Qiuping Liu^1^, Leitao Tan^1^, Qingbei Weng^1, 2*^

ORCiD: [wqs288@126.com](mailto:zhengfang03@126.com)

^#^These authors contributed equally to this work.

**Affiliations**

^1^School of Life Sciences, Guizhou Normal University, Guiyang 550025, PR China;

^2^Qiannan Normal College for Nationalities, Duyun 558000, PR China

# Supplementary Material


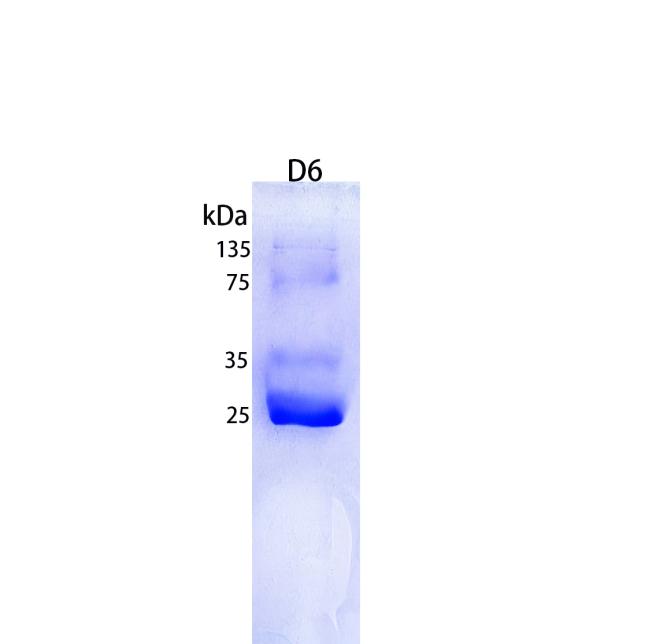


Supplementary Figure S1. SDS-PAGE analysis of the phage D6 proteins

Supplementary Table S1. List of bacterial strains and their sensitivity to phage D6

| **Bacterial strain** | **Sensitivity to D6** | **EOP** |
| --- | --- | --- |
| *Pseudomonas baetica* M-W-24 | + | - |
| *Pseudomonas frederiksbergensis* M-W-31 | + | - |
| *Pseudomonas putida* A-W-17 | + | - |
| *Pseudomonas soli* L-S-12-13 | + | - |
| *Pseudomonas fluorescens* KJ11 | + | - |
| *Pseudomonas donghuensis* L-S-(16)-2 | + | - |
| *Pseudomonas hutmensis* L-S-(8) | － | - |
| *Pseudomonas alkylphenolica* A-W-17-2 | － | - |
| *Pseudomonas migulae* N-J-48 | － | - |
| *Pseudomonas tructae* N-W-1 | － | - |
| *Pseudomonas gessardii* L-J-3 | － | - |

+: have plaques, −: no plaques after infection with phage

Supplementary Table S2. Predicted known functional proteins in phage D6

| Group | ORF | Start Codon | Start | Stop | Length | strand | Predicted function | Evalue | Identity  (%) | Accession |
| --- | --- | --- | --- | --- | --- | --- | --- | --- | --- | --- |
| Structure | 71 | ATG | 45504 | 44956 | 549 | - | major head protein | 2.00E-63 | 61.62 | YP_010347902.1 |
|  | 128 | ATG | 73456 | 73040 | 417 | - | virion structural protein | 7.00E-87 | 86.96 | YP_010347843.1 |
|  | 158 | ATG | 93090 | 95039 | 1950 | + | virion structural protein | 0 | 50.53 | YP_001957019.1 |
|  | 181 | ATG | 114216 | 107215 | 7002 | - | tail fiber protein | 0 | 73.68 | YP_010347795.1 |
|  | 186 | ATG | 122248 | 122961 | 714 | + | virion structural protein | 5.00E-165 | 93.67 | YP_010347789.1 |
|  | 187 | ATG | 122973 | 123809 | 837 | + | head maturation protease | 3.00E-179 | 86.69 | YP_010347788.1 |
|  | 201 | ATG | 132076 | 132945 | 870 | + | baseplate assembly protein | 0 | 82.29 | YP_010347772.1 |
|  | 203 | ATG | 136193 | 134436 | 1758 | - | virion structural protein | 0 | 71.19 | YP_010347769.1 |
|  | 208 | ATG | 141385 | 140072 | 1314 | - | virion structural protein | 0 | 83.30 | YP_010347764.1 |
|  | 213 | ATG | 145124 | 144258 | 867 | - | virion structural protein | 4.00E-154 | 72.57 | YP_010347759.1 |
|  | 217 | ATG | 147646 | 148314 | 669 | + | virion structural protein | 1.00E-151 | 92.79 | YP_010347754.1 |
|  | 221 | ATG | 154478 | 150363 | 4116 | - | virion structural protein | 0 | 63.26 | YP_010347750.1 |
|  | 222 | ATG | 155145 | 154609 | 537 | - | virion structural protein | 2.00E-60 | 61.58 | YP_010347749.1 |
|  | 223 | ATG | 155762 | 155169 | 594 | - | tail sheath protein | 2.00E-109 | 78.46 | YP_010347748.1 |
|  | 224 | ATG | 156567 | 155791 | 777 | - | tail fiber protein | 1.00E-144 | 79.07 | YP_010347747.1 |
|  | 225 | ATG | 157116 | 156577 | 540 | - | virion structural protein | 2.00E-102 | 82.02 | YP_010347746.1 |
|  | 230 | ATG | 159860 | 158961 | 900 | - | virion structural protein | 0 | 89.26 | YP_010347741.1 |
|  | 234 | ATG | 162747 | 161419 | 1329 | - | virion structural protein | 0 | 62.58 | YP_010347737.1 |
|  | 235 | ATG | 164138 | 162747 | 1392 | - | virion structural protein | 0 | 64.24 | YP_010347736.1 |
|  | 236 | ATG | 165501 | 164146 | 1356 | - | virion structural protein | 0 | 66.30 | YP_010347735.1 |
|  | 238 | ATG | 168197 | 165876 | 2322 | - | virion structural protein | 0 | 66.93 | YP_010347733.1 |
|  | 239 | ATG | 168308 | 169558 | 1251 | + | virion structural protein | 0 | 86.54 | YP_010347732.1 |
|  | 240 | ATG | 172311 | 169597 | 2715 | - | virion structural protein | 0 | 87.60 | YP_010347731.1 |
|  | 241 | ATG | 172345 | 174507 | 2163 | + | virion structural protein | 0 | 86.94 | YP_010347730.1 |
|  | 242 | ATG | 174516 | 175394 | 879 | + | virion structural protein | 4.00E-127 | 61.64 | YP_010347729.1 |
|  | 243 | ATG | 175402 | 175827 | 426 | + | virion structural protein | 8.00E-41 | 51.75 | YP_010347728.1 |
|  | 252 | ATG | 186150 | 185605 | 546 | - | virion structural protein | 5.00E-117 | 89.50 | YP_010347718.1 |
|  | 307 | ATG | 208047 | 206650 | 1398 | - | virion structural protein | 0 | 89.89 | YP_010347662.1 |
|  | 309 | ATG | 210062 | 208635 | 1428 | - | virion structural protein | 0 | 88.42 | YP_010347660.1 |
|  | 311 | ATG | 213914 | 211761 | 2154 | - | virion structural protein | 0 | 71.19 | YP_010347658.1 |
|  | 312 | ATG | 215180 | 214017 | 1164 | - | Internal head protein | 0 | 69.77 | YP_010347657.1 |
|  | 313 | ATG | 216721 | 215225 | 1497 | - | Internal head protein | 0 | 70.74 | YP_010347656.1 |
|  | 314 | ATG | 219279 | 216814 | 2466 | - | Internal head protein | 6.00E-175 | 41.63 | YP_010347655.1 |
|  | 316 | ATG | 222095 | 220971 | 1125 | - | putative structural head protein | 2.00E-115 | 50.94 | YP_010347653.1 |
|  | 317 | ATG | 223192 | 222098 | 1095 | - | virion structural protein | 7.00E-172 | 65.00 | YP_010347652.1 |
|  | 318 | ATG | 224504 | 223176 | 1329 | - | virion structural protein | 0 | 77.63 | YP_010347651.1 |
|  | 319 | ATG | 225034 | 224513 | 522 | - | virion structural protein | 2.00E-111 | 86.13 | YP_010347650.1 |
|  | 320 | ATG | 225936 | 225046 | 891 | - | virion structural protein | 0 | 83.62 | YP_010347649.1 |
|  | 321 | ATG | 227178 | 225952 | 1227 | - | virion structural protein | 0 | 82.81 | YP_010347648.1 |
|  | 322 | ATG | 227215 | 228300 | 1086 | + | virion structural protein | 0 | 85.04 | YP_010347647.1 |
|  | 323 | ATG | 228297 | 231197 | 2901 | + | virion structural protein | 0 | 84.47 | YP_010347646.1 |
|  | 324 | ATG | 232523 | 231234 | 1290 | - | virion structural protein | 0 | 66.82 | YP_010347645.1 |
|  | 325 | ATG | 232959 | 232534 | 426 | - | virion structural protein | 5.00E-82 | 80.85 | YP_010347643.1 |
|  | 326 | ATG | 234227 | 232986 | 1242 | - | virion structural protein | 0 | 77.67 | YP_010347642.1 |
|  | 330 | ATG | 238611 | 237367 | 1245 | - | virion structural protein | 3.00E-99 | 43.84 | YP_001957175.1 |
|  | 334 | ATG | 243386 | 242526 | 861 | - | putative structural head protein | 9.00E-149 | 71.68 | YP_010347635.1 |
|  | 392 | ATG | 288725 | 287958 | 768 | - | virion structural protein | 1.00E-151 | 77.95 | YP_010347568.1 |
|  | 395 | ATG | 290179 | 291426 | 1248 | + | putative head structural protein | 0 | 91.57 | YP_010347565.1 |
|  | 399 | ATG | 294193 | 293324 | 870 | - | virion structural protein | 0 | 97.23 | YP_010347561.1 |
|  | 400 | ATG | 296327 | 294231 | 2097 | - | tail sheath protein | 0 | 93.37 | YP_010347560.1 |
|  | 401 | ATG | 296454 | 297389 | 936 | + | virion structural protein | 0 | 84.89 | YP_010347559.1 |
|  | 402 | ATG | 297402 | 300116 | 2715 | + | virion structural protein | 0 | 82.41 | YP_010347558.1 |
|  | 403 | ATG | 300116 | 301801 | 1686 | + | virion structural protein | 0 | 89.48 | YP_010347557.1 |
| Packaging | 251 | ATG | 185546 | 183321 | 2226 | - | major capsid protein | 0 | 92.70 | YP_010347719.1 |
|  | 404 | ATG | 301933 | 304089 | 2157 | + | terminase large subunit | 0 | 89.90 | YP_010347556.1 |
| DNA replication  and modification | 1 | ATG | 645 | 2915 | 2271 | + | ribonucleotide reductase of class Ia (aerobic), alpha subunit | 0 | 63.30 | QEM42118.1 |
|  | 2 | ATG | 3003 | 4178 | 1176 | + | ribonucleoside reductase | 1.00E-170 | 60.51 | WP_015969115.1 |
|  | 7 | ATG | 8901 | 9296 | 396 | + | putative nucleoside 2-deoxyribosyltransferase | 6E-72 | 79.84 | YP_010347963.1 |
|  | 18 | ATG | 15846 | 16568 | 723 | + | HNH endonuclease | 5.00E-131 | 72.08 | YP_010347951.1 |
|  | 22 | ATG | 19372 | 20265 | 894 | + | ribosomal RNA large subunit methyltransferase N | 3.00E-163 | 75.00 | YP_010347947.1 |
|  | 25 | ATG | 21462 | 22724 | 1263 | + | RtcB protein | 0 | 82.62 | YP_010347945.1 |
|  | 52 | ATG | 37096 | 36719 | 378 | - | putative homing endonuclease | 3.00E-42 | 56.00 | YP_010347918.1 |
|  | 107 | ATG | 60847 | 59543 | 1305 | - | putative RNA ligase | 0 | 71.78 | YP_010347861.1 |
|  | 113 | ATG | 64219 | 62819 | 1401 | - | thymidylate synthase | 2.00E-168 | 54.32 | YP_010347855.1 |
|  | 115 | ATG | 66718 | 64895 | 1824 | - | nicotinamide phosphoribosyltransferase | 0 | 72.30 | YP_010347853.1 |
|  | 116 | ATG | 67669 | 66728 | 942 | - | ribose-phosphate pyrophosphokinase | 2.00E-80 | 46.37 | YP_010347852.1 |
|  | 118 | ATG | 69400 | 68021 | 1380 | - | cell division protein | 0 | 66.45 | YP_010347850.1 |
|  | 140 | TTG | 81880 | 79826 | 2055 | - | NAD-dependent DNA ligase | 0 | 73.85 | YP_010347834.1 |
|  | 142 | ATG | 83048 | 82422 | 627 | - | deoxycytidine triphosphate deaminase | 9.00E-121 | 78.37 | YP_010347832.1 |
|  | 153 | ATG | 88677 | 90800 | 2124 | + | SNF2-domain helicase | 0 | 80.75 | YP_010347820.1 |
|  | 169 | ATG | 100492 | 99428 | 1065 | - | thymidylate kinase | 0 | 73.82 | YP_010347806.1 |
|  | 182 | ATG | 114270 | 115922 | 1653 | + | putative RNA polymerase beta/ beta' subunit | 0 | 91.82 | YP_010347794.1 |
|  | 183 | ATG | 115926 | 120401 | 4476 | + | putative RNA polymerase subunit beta | 0 | 85.95 | YP_010347793.1 |
|  | 200 | TTG | 131993 | 129501 | 2493 | - | SbcCD protein, subunit C | 0 | 77.86 | YP_010347773.1 |
|  | 211 | ATG | 142347 | 143795 | 1449 | + | ribonuclease H | 0 | 92.74 | YP_010347761.1 |
|  | 214 | ATG | 145223 | 146647 | 1425 | + | putative UvsX protein | 0 | 82.62 | YP_010347758.1 |
|  | 229 | ATG | 158958 | 158314 | 645 | - | holliday junction resolvase | 4.00E-126 | 87.24 | YP_010347742.1 |
|  | 253 | ATG | 186280 | 187812 | 1533 | + | putative helicase | 0 | 94.31 | YP_010347717.1 |
|  | 269 | ATG | 194487 | 193948 | 540 | - | putative bifunctional (p)ppGpp synthetase/guanosine-3',5'-bis(diphosphate) 3'-pyrophosphohydrolase | 6.00E-58 | 57.56 | RWB08829.1 |
|  | 295 | ATG | 203262 | 202789 | 474 | - | macro domain-containing protein | 8.00E-96 | 84.71 | YP_010347672.1 |
|  | 333 | ATG | 242539 | 241193 | 1347 | - | putative RNA polymerase beta subunit | 0 | 84.60 | YP_010347636.1 |
|  | 343 | TTG | 249246 | 247789 | 1458 | - | putative RAD2/SF2 helicase | 0 | 89.90 | YP_010347626.1 |
|  | 344 | ATG | 251347 | 249371 | 1977 | - | putative RNA polyerase beta prime subunit | 0 | 91.95 | YP_010347625.1 |
|  | 345 | TTG | 253493 | 251355 | 2139 | - | putative RNA polymerase subunit beta | 0 | 91.15 | YP_010347624.1 |
|  | 352 | ATG | 261524 | 260385 | 1140 | - | nuclease SbcCD, subunit D | 0 | 87.34 | YP_010347616.1 |
|  | 360 | TTG | 266881 | 265412 | 1470 | - | RNA polymerase beta subunit | 0 | 93.25 | YP_010347607.1 |
|  | 367 | ATG | 274014 | 273196 | 819 | - | Nucleotidyltransferase | 5.00E-127 | 66.04 | YP_010347598.1 |
|  | 368 | ATG | 276161 | 274014 | 2148 | - | DNA polymerase | 0 | 89.93 | YP_010347597.1 |
|  | 383 | ATG | 283574 | 282834 | 741 | - | putative dual-specificity phosphatase | 7.00E-92 | 55.51 | YP_010347578.1 |
|  | 384 | ATG | 284572 | 283625 | 948 | - | tubulin PhuZ | 6e-180 | 80.95 | YP_010347576.1 |
| Biosynthesis | 19 | ATG | 17592 | 16657 | 936 | - | galactose oxidase | 2.00E-27 | 30.74 | MBD0355842.1 |
|  | 409 | ATG | 306531 | 306049 | 483 | - | dihydrofolate reductase | 5.00E-54 | 52.50 | YP_010347553.1 |

Supplementary Table S3. The codon usage in the phage D6 and *Pst* DC3000

| **Phage D6** | | | | ***Pst* DC3000** | | | |
| --- | --- | --- | --- | --- | --- | --- | --- |
| **AmAcid** | **Codon** | **Number** | **RSCU** | **AmAcid** | **Codon** | **Number** | **RSCU** |
| Ala | GCG | 1122 | 0.80 | Ala | GCG | 64866 | 1.14 |
|  | GCA | 1681 | 1.21 |  | GCA | 52970 | 0.93 |
|  | GCT | 1625 | 1.17 |  | GCT | 46187 | 0.81 |
|  | GCC | 1149 | 0.82 |  | GCC | 62683 | 1.11 |
| Cys | TGT | 1779 | 1.02 | Cys | TGT | 24708 | 0.64 |
|  | TGC | 1705 | 0.98 |  | TGC | 52273 | 1.36 |
| Asp | GAT | 2099 | 1.20 | Asp | GAT | 34979 | 1.00 |
|  | GAC | 1410 | 0.80 |  | GAC | 34709 | 1.00 |
| Glu | GAG | 1249 | 0.78 | Glu | GAG | 24769 | 0.85 |
|  | GAA | 1941 | 1.22 |  | GAA | 33543 | 1.15 |
| Phe | TTT | 1623 | 0.81 | Phe | TTT | 27815 | 0.91 |
|  | TTC | 2360 | 1.19 |  | TTC | 33507 | 1.09 |
| Gly | GGG | 901 | 0.72 | Gly | GGG | 27898 | 0.74 |
|  | GGA | 1350 | 1.08 |  | GGA | 23517 | 0.62 |
|  | GGT | 1766 | 1.41 |  | GGT | 36450 | 0.96 |
|  | GGC | 977 | 0.78 |  | GGC | 63234 | 1.67 |
| His | CAT | 2013 | 1.09 | His | CAT | 30942 | 0.95 |
|  | CAC | 1679 | 0.91 |  | CAC | 33924 | 1.05 |
| Ile | ATA | 1230 | 0.77 | Ile | ATA | 12762 | 0.55 |
|  | ATT | 1421 | 0.89 |  | ATT | 20751 | 0.89 |
|  | ATC | 2158 | 1.35 |  | ATC | 36063 | 1.55 |
| Lys | AAG | 1654 | 1.17 | Lys | AAG | 29467 | 1.02 |
|  | AAA | 1164 | 0.83 |  | AAA | 28370 | 0.98 |
| Leu | TTG | 2261 | 1.43 | Leu | TTG | 35550 | 1.29 |
|  | TTA | 1400 | 0.88 |  | TTA | 10483 | 0.38 |
|  | CTG | 1659 | 1.05 |  | CTG | 57074 | 2.08 |
|  | CTA | 902 | 0.57 |  | CTA | 9366 | 0.34 |
|  | CTT | 2236 | 1.41 |  | CTT | 28293 | 1.03 |
|  | CTC | 1046 | 0.66 |  | CTC | 24244 | 0.88 |
| Met | ATG | 1786 | 1.00 | Met | ATG | 31956 | 1.00 |
| Asn | AAT | 1309 | 0.76 | Asn | AAT | 20275 | 0.79 |
|  | AAC | 2156 | 1.24 |  | AAC | 30960 | 1.21 |
| Pro | CCG | 1205 | 0.92 | Pro | CCG | 50378 | 1.32 |
|  | CCA | 2135 | 1.64 |  | CCA | 42101 | 1.10 |
|  | CCT | 994 | 0.76 |  | CCT | 31651 | 0.83 |
|  | CCC | 884 | 0.68 |  | CCC | 28338 | 0.74 |
| Gln | CAG | 2244 | 1.18 | Gln | CAG | 53966 | 1.21 |
|  | CAA | 1554 | 0.82 |  | CAA | 35205 | 0.79 |
| Arg | AGG | 1212 | 0.82 | Arg | AGG | 31149 | 0.76 |
|  | AGA | 1568 | 1.06 |  | AGA | 21781 | 0.53 |
|  | CGG | 1242 | 0.84 |  | CGG | 48774 | 1.19 |
|  | CGA | 2176 | 1.47 |  | CGA | 46005 | 1.12 |
|  | CGT | 1648 | 1.12 |  | CGT | 34887 | 0.85 |
|  | CGC | 1010 | 0.68 |  | CGC | 64229 | 1.56 |
| Ser | AGT | 1675 | 0.93 | Ser | AGT | 19657 | 0.60 |
|  | AGC | 1819 | 1.01 |  | AGC | 45709 | 1.40 |
|  | TCG | 1970 | 1.09 |  | TCG | 47047 | 1.44 |
|  | TCA | 2196 | 1.22 |  | TCA | 38434 | 1.18 |
|  | TCT | 1791 | 0.99 |  | TCT | 21512 | 0.66 |
|  | TCC | 1356 | 0.75 |  | TCC | 23608 | 0.72 |
| Thr | ACG | 2011 | 1.12 | Thr | ACG | 34839 | 1.18 |
|  | ACA | 1714 | 0.95 |  | ACA | 25054 | 0.85 |
|  | ACT | 1510 | 0.84 |  | ACT | 20168 | 0.69 |
|  | ACC | 1962 | 1.09 |  | ACC | 37691 | 1.28 |
| Val | GTG | 1515 | 0.87 | Val | GTG | 34498 | 1.19 |
|  | GTA | 1819 | 1.04 |  | GTA | 16415 | 0.57 |
|  | GTT | 2188 | 1.25 |  | GTT | 30333 | 1.05 |
|  | GTC | 1454 | 0.83 |  | GTC | 34389 | 1.19 |
| Trp | TGG | 1923 | 1.00 | Trp | TGG | 42568 | 1.00 |
| Tyr | TAT | 950 | 0.68 | Tyr | TAT | 12989 | 0.86 |
|  | TAC | 1833 | 1.32 |  | TAC | 17068 | 1.14 |
| End | TGA | 1847 | 1.35 | End | TGA | 38177 | 2.00 |
|  | TAG | 1189 | 0.87 |  | TAG | 9062 | 0.47 |
|  | TAA | 1062 | 0.78 |  | TAA | 10105 | 0.53 |

Supplementary Table S4. The tRNAs availability analysis in the phage D6

| **Number** | **tRNA** | **tRNA Begin** | **tRNA End** | **tRNA Type** | **Anticodon** | **Length** | **Isotype Score** |
| --- | --- | --- | --- | --- | --- | --- | --- |
| 1 | GTGAACATAGTTCAGTTGGCAGAACCCCAGGATGTGACCCTGGTGGTCGGGAGTTCGATCCTC CCTGTTCACA | 9948 | 10020 | His | GTG | 73 | 70.9 |
| 2 | GGCTCCATAGCGCAGTTGGTTAGCGCACCGGCCTGTCACGCCGGGGGTCAGGGGTTCAAGTCCCCTTGGAGTCG | 10029 | 10105 | Asp | GTC | 76 | 84 |
| 3 | GGGTTGTTAGGGGAGCGGTCAAACCCATCGGACTGTAAATCCGCGCCTATGGCTACCGTGGTTCGAATCCACGCCAACCCA | 10251 | 10334 | Tyr | GTA | 84 | 53.3 |
| 4 | CAGCGTGTAGTTCAGTTGGTAGAATGCATGGTTTGGGACCATGTGGTCGTAGGTTCGAAGCCTACCACTCTGA | 10449 | 10524 | Pro | TGG | 76 | 57.5 |
| 5 | CCCACCTTAGCTTAGTTGGGAAAGCGCATCCATCGTCGCTGTACGTTG GATGAGACACGGGTTCGATCCCCGTAGGTAGGG | 10533 | 10613 | Thr | CGT | 81 | 38.3 |
| 6 | TTCGAGATAGCTCAGTTGGTAGAGCAGCGGACTGTTAATCCGTTGGTCCCAGGTTCGAACCCTGGTCTCGGAG | 10814 | 10889 | Asn | GTT | 76 | 78.2 |
| 7 | TCCCACTTAGCTCAGTTGGTAGAGCACACGGCTGTTAACCGTGCGGTCCCTGGTTCGAGTCCAGGAGTGGGAG | 10895 | 10970 | Asn | GTT | 76 | 80.2 |
| 8 | GGGTCATTAGTTCAATTGGCAGAACACCGGCCTCCAAAGCCGGGTGTTGGTGGTTCGAGTCCATCATGGCCCG | 14813 | 14888 | Trp | CCA | 76 | 81.1 |
| 9 | GGTGAAATGGTAGAGTGGTTTAATACATCCCCTTGCTAAGGGGACGGGTCTTCGAAGGCCCCCTGGGTTCAAATCCCAGTTTCACCT | 14895 | 14984 | Ser | GCT | 90 | 68.2 |
| 10 | GTCAGAATGATCGAATTGGTATAGATAGCTCACTTAAAATGAGCCGCCTTCGGGATTGGTGGGTTCGAGTCCCCCTTCTGACA | 15691 | 15776 | Leu | TAA | 86 | 53.8 |
| 11 | GCGGATGTGGCGAAATTGGTAGACGCGCTGGATTTAGGTTCCAGTGGGCTTGCTCGT GGGAGTTCGAGTCTCCCTATCCGCA | 17654 | 17738 | Leu | TAG | 85 | 67.8 |
| 12 | GGAGAGGTGCGTGAGTGGTTGTAAACGAGCGGTCTTGAAAACCGCCAGGTGTAAAAGCCTCCAGGGTTCGAATCCCTGTCTCTCCG | 18744 | 18832 | Ser | TGA | 89 | 73.9 |
| 13 | GCTGAGATAGCAAAGTGGTCGAATGCACGGGATTGCAAATCCTGTTTCTTAAGAACATCGGGGGTTCAAATCCCTCTCTCAGCT | 18840 | 18926 | Cys | GCA | 87 | 65.6 |
| 14 | GCCGCTATAGCTCAGTTGGTAGAGCAGCTGACTTGTAATCAGCAGGTCCACAGTTCGAATCTGTGTGGCGGCA | 18928 | 19003 | Thr | TGT | 76 | 86.5 |
